# Recent advances in enhances peripheral nerve orientation: the synergy of micro or nano patterns with therapeutic tactics

**DOI:** 10.1186/s12951-024-02475-8

**Published:** 2024-04-20

**Authors:** Majid Sharifi, Mohammad Kamalabadi-Farahani, Majid Salehi, Somayeh Ebrahimi-Barough, Morteza Alizadeh

**Affiliations:** 1 Student Research Committee, School of Medicine, Shahroud University of Medical Sciences, Shahroud, Iran; 2 Department of Tissue Engineering, School of Medicine, Shahroud University of Medical Sciences, Shahroud, Iran; 3 Department of Tissue Engineering and Applied Cell Sciences, School of Advanced Technologies in Medicine, Tehran University of Medical Sciences, Tehran, Iran; 4 Department of Tissue Engineering and Biomaterials, School of Advanced Medical Sciences and Technologies, Hamadan University of Medical Sciences, Hamadan, Iran

**Keywords:** Nerve regeneration, Cell arrangements, Micro- or Nano-patterns, Electrical stimulation, Conduits

## Abstract

Several studies suggest that topographical patterns influence nerve cell fate. Efforts have been made to improve nerve cell functionality through this approach, focusing on therapeutic strategies that enhance nerve cell function and support structures. However, inadequate nerve cell orientation can impede long-term efficiency, affecting nerve tissue repair. Therefore, enhancing neurites/axons directional growth and cell orientation is crucial for better therapeutic outcomes, reducing nerve coiling, and ensuring accurate nerve fiber connections. Conflicting results exist regarding the effects of micro- or nano-patterns on nerve cell migration, directional growth, immunogenic response, and angiogenesis, complicating their clinical use. Nevertheless, advances in lithography, electrospinning, casting, and molding techniques to intentionally control the fate and neuronal cells orientation are being explored to rapidly and sustainably improve nerve tissue efficiency. It appears that this can be accomplished by combining micro- and nano-patterns with nanomaterials, biological gradients, and electrical stimulation. Despite promising outcomes, the unclear mechanism of action, the presence of growth cones in various directions, and the restriction of outcomes to morphological and functional nerve cell markers have presented challenges in utilizing this method. This review seeks to clarify how micro- or nano-patterns affect nerve cell morphology and function, highlighting the potential benefits of cell orientation, especially in combined approaches.

**Graphical Abstract:**

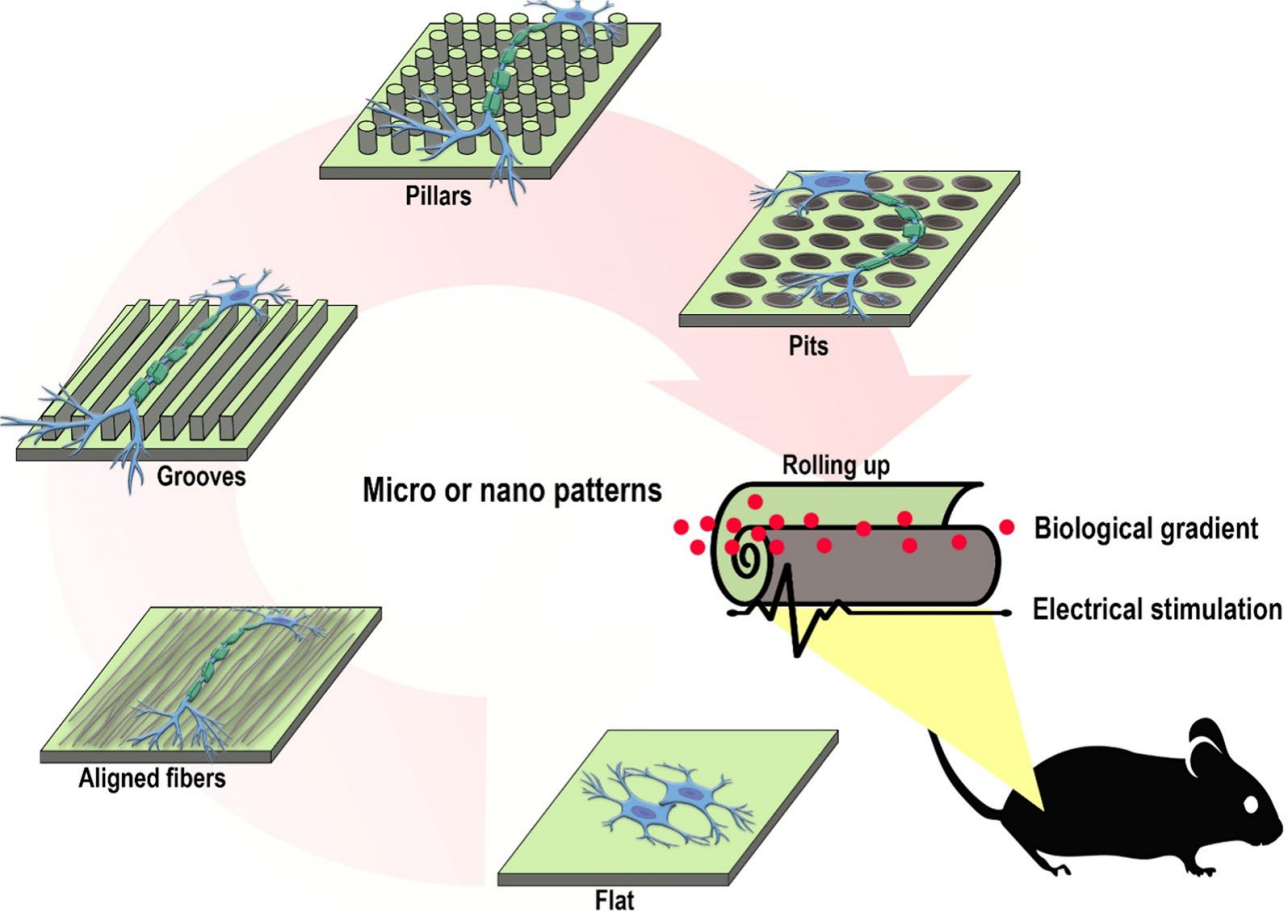

## Introduction

Low and non-uniform cell density, disorganization in nerve cell arrangements like nerve coils, and instability of seeded cells can result in sensory-motor problems in healing peripheral nerve tissues [1]. Therefore, rapid repair of neural tissue with grafts or conduits is not the only treatment option. Regenerative methods should prioritize the seeding site, proliferation, differentiation, and cell shape for cell orientation and alignment to ensure that developing neurons resemble natural structures [2]. Alignment and orientation of neurons during the repair process have been demonstrated to have a positive impact on the function of neural tissue, including increased NCV, reduced neuroma, and improved CMAP [3–5].

Nanomedicine-based methods allow tissue engineering to regulate the topography [6], biological or chemical gradients of the microenvironment [7], and provide electrical or mechanical stimulation in three-dimensional space [8] for stimulating neurons during treatment. Scaffold or conduit surfaces have been demonstrated to influence cell orientation and alignment during cell proliferation, growth, and development through topological nanostructures such as grooves, pillars, and pits [9]. Conduits also enable diverse cellular interactions to orient cells by controlling the three-dimensional space containing biological or chemical gradients induced by nanocarriers [7]. It seems that incorporating topological nanostructures and biological or chemical gradients into conduits not only more effectively controls neuronal migration but also improves cell adhesion and signal transduction, facilitating the direction of axonal growth in an injured environment. Despite significant physicochemical similarities between luminal surfaces and the natural microenvironment, variations in geometry, topological patterns, and diverse mechanisms of cell contact with surfaces can lead to different outcomes. The regenerative consequences of these differences are not fully understood.

Biological or chemical gradients and topological strategies derived from micro- or nano-carriers were found to be important for the alignment, orientation, and migration of neurons during repair. Neurons guide the growth cone and directional elongation of neurites/axons toward secreted signaling molecules such as NGF and Sema3A [10]. Similar to neurotrophic factors, chemotropic factors have been demonstrated to effectively control cell orientation by regulating neurite growth based on their source of release [11]. In contrast to topological micro- or nano-patterns, diverse synergistic effects of neurotrophic and chemotrophic factors are provided to induce neuronal orientation [10–12]. However, controlling signal transduction mechanisms, managing diffusion within 3D structures, enhancing cellular responses such as migration and cell morphology development, and addressing the ambiguous responses of polarized cells to sudden reversal gradients pose challenges to this approach. Gradients-induced cell polarization can increase the likelihood of cell migration in the short or long period [13]. Thus, the accumulation of cells at high pressure gradients is a significant issue. Modeling gradients based on topological micro- or nano-patterns seems to address the challenges of cell migration in 3D structures. The diverse impacts of biological or chemical gradients on cell survival add complexity to the comprehension of cell behavior and functional responses.

In addition to topological micro or nano structures and biological or chemical gradients, neural tissue has been shown to exhibit significant repair responses when exposed to electrical stimulation with voltages below 20 Hz [14]. Despite the importance of internal electrical signals in nerve healing through pain relief [15], the use of external electric fields in repairing injured nerves by promoting re-innervation and neurites/axons orientation also has considerable potential [16]. Electrical stimulation can affect nerve cells and cause biochemical reactions. These reactions can be exploited to enhance axonal sprouting and spreading toward stimuli by modifying membrane proteins such as ATPase pumps and ion transport channels [17]. Four effects of electrical stimulation on neurons have been identified: (i) alterations in the movement and concentration profile of cytoplasmic molecules, (ii) modifications in membrane response due to changes in transmembrane potential, (iii) electrophoretic accumulation of surface molecules, and (iv) heightened secretion of neurotrophic factors [15, 18, 19]. However, the mechanism of the effect of electrical stimulation on cell orientation or polarization is still not fully understood. Conflicting results from electrical stimulation may be due to the injury type, stimulation type and location, frequency and duration of stimulation, treatment strategy (simple or combined), and measured metrics.

Despite the notable advances in strategies involving topography, biological/chemical gradients, and electrical stimulations for PNI regeneration, the individual application of these methods has not fully met therapeutic expectations, particularly in cases of extensive injuries [20]. Hence, the exploration of synergistic approaches that mimic the natural interactions of tissues with micro- or nano-patterns, along with biological/chemical gradients, holds significant promise for neural tissue regeneration. For instance, despite the effective use of conduits with micro- or nano-patterns for mechanical support and targeted signal amplification for cell adhesion and directional neurites/axons growth [21, 22], they remain ineffective due to the absence of necessary temporal and spatial adjustments needed by neurites when connecting proximal and distal ends. Releasing neurotrophic factors at specific times according to gradients can offer a more precise expansion pattern facilitating the connection of the two nerve ends [23]. Also, enhancing nerve cell performance through electrical signals in conduits with micro- or nano-patterns effectively accelerates regeneration speed and process [24]. This study aims to illuminate the synergistic effects of therapeutic approaches employing micro and nano patterns on the directional growth of neurites/axons and cell orientation.

## Structural characteristics and cellular interactions of aligned nervous tissue

Peripheral nerve tissue has aligned and organized cellular structures, similar to muscles, tendons, the digestive system, and the heart [25]. Adhering to natural neuronal structural patterns during regeneration enhances performance and reliability, based on insights from tissue regeneration. This objective has not been accomplished yet due to the distinct responses of cells to mechanical and biochemical stimuli. Recognizing structural patterns in neural tissue and cell behavior, and choosing regeneration methods based on these patterns, could enhance the chances of aligning and orienting neurons.

### Anatomy

Peripheral nerves are groups of nerve fibers, axons or dendrites that are surrounded by connective tissue with sensory or motor characteristics or a mixture of them [26]. These layers are the endoneurium, which is flexible and longitudinally connected to capillaries, the perineurium, a thin, dense barrier that acts as a blood barrier, and the epineurium layer, which offers strong mechanical protection. The axon cytoskeleton is made up of microfilaments, microtubules, and intermediate filaments [27]. It plays a crucial role in maintaining axon structure, migration, attachment, and growth. Along with the cytoskeleton, the abundant P0 protein is also important as it mediates between the axon and myelin sheath produced by Schwann cells [28]. Myelin is essential for insulating axons and transmitting electrical signals, but it may also contribute to neuronal tensile strength, growth, and development by generating connectors like the ECM and integrin [29]. Also, despite the variation in mechanical properties of peripheral nerves depending on the type and location, the overall regeneration of the peripheral nerve system is influenced by factors like elastic modulus, ultimate tensile strength, and elongation at break of the respective nerve [30]. These properties impact the alignment and orientation of neurons by transmitting mechanical forces.

### Cell attachment

**Fig. 1:**
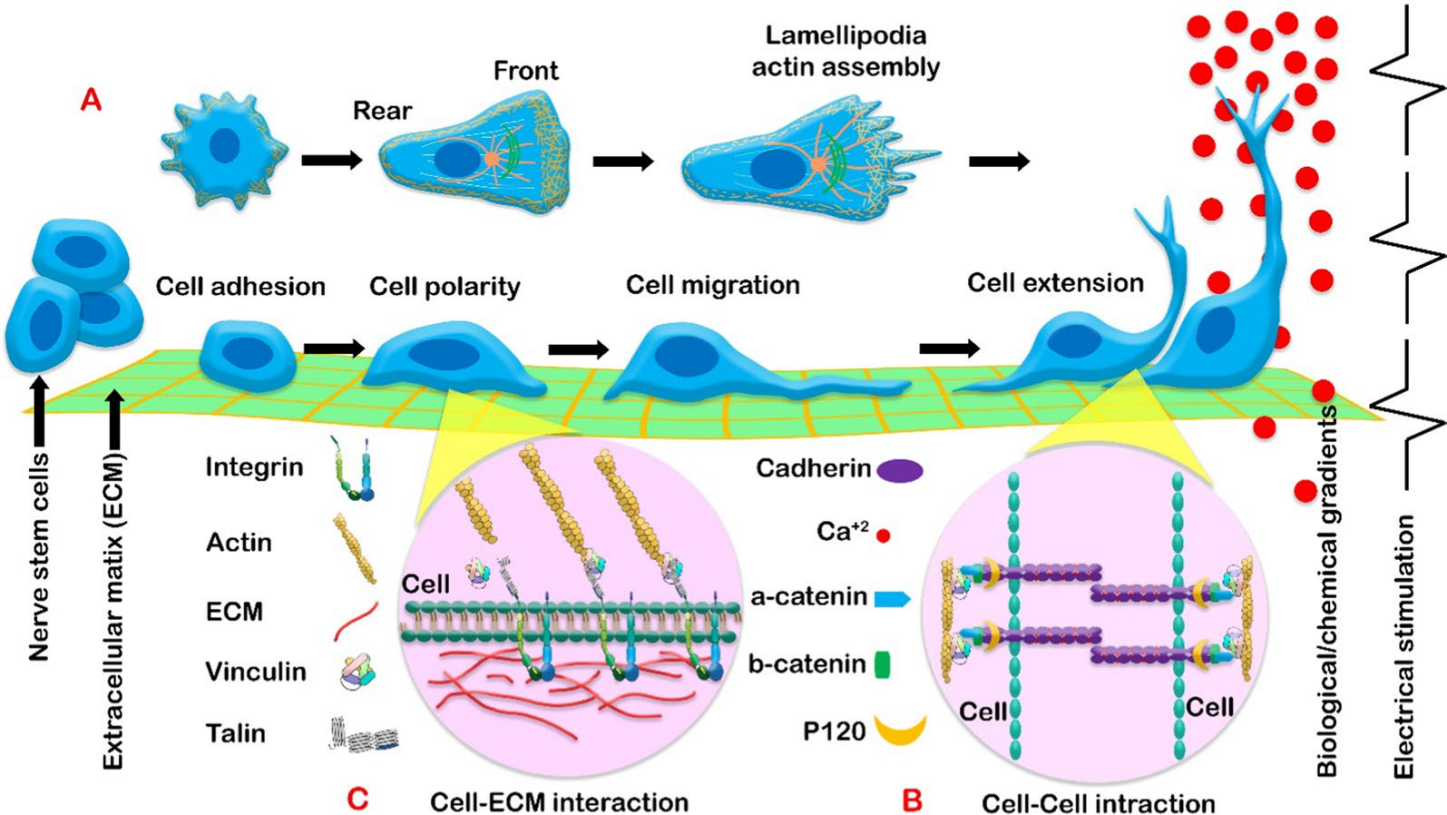
Schematic view of cellular function in conduits or scaffolds: **A** Cellular responses to environmental influences can be studied by controlling changes in the morphological properties and molecular structure of the cytoplasm and nucleus. Key indicators comprise of (1) adhesion and migration, which are based on the accumulation of intracellular actin and formation of filopodia, and (2) cell orientation or elongation in response to internal stimuli (biological/chemical gradient) or external stimuli (electrical impulses). **B** The cadherin/catenin complex is involved in cell–cell interaction, that E-Cadherin immobilizes synthesized β-catenin to the nerve cell membrane. Meanwhile α-catenin links β-catenin to the actin cytoskeleton. **C** Diagram of nerve Cell-ECM interaction displaying the sequence of main receptors and intra- and extracellular links

One of the criteria for measuring cell health is the assessment of cellular behavioral responses, such as proliferation, differentiation, adhesion, migration, and reactions to physical, chemical, and biological signals (Fig. 1A). Therefore, the determination of cell–cell and cell-ECM connections and their indicators may represent important aspects of the health of neuronal behavioral functions.

#### Cell–cell interaction

During growth and regeneration, neurons, like other cells, primarily receive signals from neighboring cells through the cadherin family (including classical cadherins, proto-cadherins, and atypical transmembrane protein 7-cadherins), L1, and NCAM [31]. Cadherins play important roles in cell identification, sorting, migration, and polarity via calcium ions [32]. They bind to cells through mediators such as p120 and catenins (Fig. 1B) [32]. By regulating the cytoskeletal state of neurons through Rho, Rac, and Cdc42, they affect adhesion and migration by extending projections and exploring neighboring cells [33]. Also, L1 and NCAM (types 120, 140, and 180) impact cell–cell interactions like synapse formation and axon growth by binding to other molecules such as integrin [31]. NCAM, especially NCAM-180, and L1 through FGFR, activate the downstream MAPK/ERK pathway, impacting spectrin activation and the actin cytoskeleton, thereby contributing to neuronal regeneration and cell–cell contact [34]. Additionally, neurons hinder Schwann cell migration by increasing the expression of NCAM and L1 [31]. The start of Schwann cell-axon interaction usually involves increased Neuregulin-1 in the developing axon [35]. This triggers PI3-kinase, phospholipase C, and MAPK signaling pathways in Schwann cells, resulting in neuronal cell growth, migration, and myelination.

#### Cell-ECM interaction

The ECM is essential as the main structural support in the body, performing specialized functions tailored to the needs of each organ. Despite its diverse composition, collagen, elastin, fibronectin, and laminin are the main components of the ECM [36]. Collagen is plentiful in peripheral nerve tissue, which is associated with the layer formed by Schwann cells [36]. The ECM plays a crucial role in regulating neuronal functions, including differentiation, migration, alignment, adhesion, cell–cell communication, and the dispersion of mature neurons. This is achieved through interactions between cell integrins and laminin, fibronectin, collagen, and ECM peptides [37]. Integrins are transmembrane receptors composed of α and β subunits that form noncovalent interactions [38]. These interactions are observed in four ways in neuron-extracellular matrix interactions: RGD (Arg-Gly-Asp) ligands bind to αV, α5β1, α8β1, and αIIbβ3 integrins, LDV (Leu-Asp-Val) ligand binds to integrins α4β1, α4β7, and α9β1, Laminin/collagen binds integrins α1, α2, α10, α11, and laminin binds integrins β1 (α3, α6, and α7) and α6β4 [38, 39]. Integrin-mediated neuron-ECM interactions facilitate two-way signaling (outside-in and inside-out) between cells and ECM (Fig. 1C). These interactions involve talin, paxillin, zyxin, and vinculin, along with actin filaments, and contribute to the formation of different adhesive structures like nascent adhesions, focal complexes, focal adhesions, and fibrillar adhesions [40]. Paxillin-rich, short-lived nascent point-like adhesions are found beneath the lamellipodia at the front of the migrating cell. Focal adhesions containing low levels of paxillin and high levels of vinculin form at the ends of stress fibers containing actin and myosin [41]. This cytoskeleton-derived adhesion is effective in force transmission. The most stable adhesions, fibrillar adhesions containing large amounts of tensin and β1-integrin, form along matrix fibrils beneath the cell center [39]. During cell migration, the cytoplasm expands by filopodia and lamellipodia, adhesions form, creating focal complexes rich in FAK and paxillin, and actin polymerization also takes place. Actomyosin contractile forces release mature focal adhesions on the opposite side, separating the cell from the environment [38, 40, 41].

## Supporting structures

Efforts have been made to develop support structures containing neural cells to alleviate the limitations of autograft and allograft in peripheral nerve regeneration, with the most important being biological or polymeric conduits. Despite the significant impact of construction methods and materials on the healing process through biocompatibility, biodegradability, permeability, and mechanical capabilities (tensile strength, pressure resistance, flexibility, etc.) (Table 1), this study specifically examines the role of neuron function (adhesion, proliferation, differentiation, growth) in the physical structure of conduits, including orientation and topology patterns. While it may be challenging to establish criteria based on the physical properties of conduits, a review of existing literature may offer valuable insights to enhance and expedite the repair process of neural tissue.

**Table 1:** A summary of the features and construction methods of conduits used in peripheral nerve repair

Item		Descriptions	Advantages	Disadvantages
A. Elements [42–48]:				
Biological conduits		• These conduits typically consist of vessels, muscles (muscle fibers), amniotic membrane, biological membranes, fats, and nerve trunks • The decellularization approach (physical, chemical, and enzymatic) is essential to minimize graft rejection when using allogeneic or xenogeneic biological conduits	Unlimited supply, reduced surgical time, high biodegradability and bio-absorption, natural topological structures and patterns, improved cell functions such as adhesion, proliferation, differentiation, and development	Unfavorable in long nerve transections, immunogenicity enhancement, rapid destruction, disease transmission, adverse structural changes during processing, ethical concerns, need to pharmacological suppressors, diverse cellular responses, and long manufacturing processes
Polymeric conduits	Natural	• The most significant natural polymers are chitosan, silk fibroin, collagen, alginate, gelatin, and hyaluronic, which collagen has garnered the most attention • Asymmetric degradation of natural polymers suggests that the resulting conduits should be less than 15 mm in length	High biodegradability and biocompatibility, coming from renewable sources, simple and cheap generation, high biological absorption, high permeability and porosity during repair, reducing inflammation in most cases, high cell adhesion, and strengthening angiogenesis	Variable mechanical properties with a low Young’s modulus, thermal and mechanical limitations in conduit production, low process ability asymmetric destruction, rapid decomposition in pH < 7, potential transmission of viral or bacterial, and heterogeneous chemical and physical structures
	Synthetic	• Among the wide range of synthesized polymer compounds, the most crucial ones for nerve repair activities are PLA, PGA, PCL, PLGA, PLLA, and PDMS • Complete compliance of synthetic polymer-based conduits with the nerve recovery period (3–6 months) due to the delay in degradation	High mechanical properties (strength, elasticity, flexibility), high thermal or chemical stability, acceptable performance in all gaps, excellent plasticity, controlled biodegradability, abundant resources, relatively simple production, chemical uniformity of structures	Weak cell adhesion, relatively high immunogenicity, limited bioactivity, poor biocompatibility, ischemia of nearby tissues, decreased angiogenesis, limited reproducibility, acidic byproducts, and low process ability
Non organic conduits		• The most important inorganic compounds in nerve tissue regeneration are silicone hydrogels and carbon compounds	High thermal or electrical conductivity, anti-bacterial, very elastic (up to ~ 18%) elongation, high tensile strength, and flexibility	High toxicity, impurities, blood clotting, immunogenicity, and long-term stability in nerve tissue
B. Fabrication methods [1, 49–52]:				
Printing methods	Extrusion	• Extrusion printing defined with injecting a molten thermoplastic polymer filament through a heated nozzle onto the printing surface • The nozzle moves in the x, y and z directions according to software instructions, and the extruded materials are injected by pneumatic pressure	Cost-effectiveness, good mechanical properties, high commercial availability, wide range of biomaterials, excellent performance with various cell types, minimal cell damage compared to other techniques, rapid conduit production, low extrusion temperature, and shape versatility	Limited filament clarity (typically over 100 µm) with minimal surface detail, increased risk of layer falling, abnormal fusion of initial layers, relatively low strength, weak end layers bonding, requirement for material of viscose, and restricted to thermoplastic materials
	Inkjet	• In this system, fine droplets of polymer solution are deposited along the x, y, and z axes to create a pattern on the substrate • To produce bio-ink droplets, it uses piezoelectric pulses (by sound waves) and thermal pulses (by electric heat)	Good resolution of 50 to 75 µm, ability to print with pL volume droplets, minimal thermal effects, no contact between nozzle and conduit, rapid gelation, high operational capability, heterogeneous multicell capability, and cell viability of over 85%	Restricted to low-viscosity materials, uncertainty cell encapsulation, clogging at high viscosity, limitations in cell density, relatively subpar mechanical properties, atypical droplet dryness, height restrictions, challenges in 3D geometry, and longtime manufacturing
	Laser	• Using a laser source to create scaffolds on substrates, which is done through laser-guided direct writing or laser-induced forward transfer • This process involves a focusing system (for alignment), a ribbon, a pulsed laser beam (for efficient transmission), and a substrate	The use of bio-ink with high viscosity, a wide range of materials, a high concentration of cells, and a system that prevents nozzle blockage, allows for fine shaping, excellent arrangement of cell patterns, high cell viability, accuracy, and rapid gelation	Longtime printing, elevated thermal impact, increased expenses, diminished mechanical resilience, and aggregation at the final construct
	Stereo-lithography	• This technology employs a light source, such as ultraviolet or infrared with a light-sensitive resin, to produce 3D structures	Highly accurate 3D shapes, and simple removal of material trapped in the scaffold	Consumable materials limitation based on photo response, high toxicity of resins, and high production cost
Textile engineering methods	Electrospinning	• Electrospinning has the characteristics of electrospraying and dry spinning • The polymer solution is injected by a low-speed syringe pump into an external electrostatic field and then collected on the collector	Simple production process, scalable, structure similar to extracellular matrix, cheap, scalable, ability to produce nanometer to micrometer fibers in one structure, high porosity, and high surface to volume ratio	Use of toxic solvents, insufficient cell filtration, asymmetric cell distribution, injection instability, non-repeating structures, the effect of environmental and voltage on the structure, and high energy consumption
	Other technology	• Woven, knitting, and braiding technologies are the primary methods used in the textile field to fabricate conduits • The potential for creating conduits layer by layer according to nerve repair requirements	High bionic surface morphology, flexible, suitable mechanical connections, low cost, easy technique, rapid production of conduits with similar size and shape, mechanical strength and pore size scalable, high cell adhesion and migration	Limited diameter of woven conduits, improper increase of wall thickness of conduits, expensive software and hardware package for adjusting the weaves direction
Decellularization methods	Physical	• Biological tubes are decellularized using freezing, hydrostatic pressure, electric currents, mechanical shocks, gamma radiation, and supercritical fluids to produce conduits	Facilitated decellularization, minimal immunogenicity, low cost, potential for commercialization	Destruction of tubular structure, low efficiency of cell removal in dense tissues, and unfavorable compaction of ECM
	Enzymatic	• Collagenase, trypsin, galactosidase, nuclease, and pepsin are enzymes that remove tissue and cell debris, often considered as an auxiliary process	Cell membrane digestion, complete elimination of antigenic and genomic compounds, no impact on ECM, and targeted removal of specific compounds	Long-term presence has a negative effect on ECM and cell membrane, complex washing, long process with undetermined time, possible immunogenicity with enzyme retention, changes in protein microstructures
	Chemical	• Direct removal of cells by washing in chemical agents such as acids (peracetic acid, H_2_SO4, etc.), alkalis (NH_4_OH, NaOH), EDTA, TnBP, SDS, ethylene oxide and acetone: ethanol, Triton X-100	Highly efficient cell removal with minimal genomic residues, preservation of tubular structure, optimal preservation of ECM structure (collagen, elastin, proteoglycans, etc.) based on the type of chemical agent, and preservation of binding factors	Challenges vary depending on the type of chemical agent, including collagen and glycosaminoglycan degradation by SDS, low cell removal efficiency with Triton X-100, low cell removal efficiency with EDTA, and toxicity of chemical agents

Ethylenediaminetetraacetic acid (EDTA), Poly co-glycolic acid (PLGA), Poly L-lactic acid (PLLA), Polycaprolactone (PCL), Polydimethylsiloxane (PDMS), Polyglycolide (PGA), Polylactide (PLA), Sodium dodecyl sulfate (SDS), Tri(n-butyl) phosphate (TnBP)

### Aligned fiber structures

Repairing injured peripheral nerves is often challenging, and sometimes even impossible, due to the misdirection of nerve cells and or improper suturing of the two nerve endings. Leveraging the inherent properties of neural tissue, such as its striped structure and directional cell distribution, conduits with aligned fibers have been found to expedite the healing process by facilitating the directional growth of neurons. Numerous studies in this area have indicated that the use of aligned fibers promotes peripheral nerve regeneration and enhances performance (Table 2). Nevertheless, achieving the precise extent and shape of cell alignment remains problematic due to the adverse effects of excessively aligned fibers on cell junctions and the unbalanced growth of axons.

**Table 2:** The performance of aligned or random fibers in the peripheral nerve regeneration

Conduit	Method	Fiber	Outcomes	Ref
PCL/CNTs	Model: In vivo Damage length: 10 mm Period: 3 months	Random	SFI: − 69.48; CMAP: 4.27 mV; myelin sheath: 0.21 µm; low expression of NF200, MBP, Tuj-1 genes; weight percentage: 36%	[53]
		Aligned	SFI: − 56.89; CMAP: 3.48 mV; myelin sheath: 0.68 µm; high expression of NF200, MBP, Tuj-1 genes; weight percentage: 46%	
Fibrin nanofibers	Model: In vivo Damage length: 10 mm Period: 12 weeks	Random	SFI: − 60.14; CMAP: 5.01 mV; myelin sheath: 50.2 µm; diameter of myelinated fibers: 309 µm; percentage of muscle fiber area: 20.5%	[54]
		Aligned	SFI: -43.19; CMAP: 6.68 mV; myelin sheath: 65.8 µm; diameter of myelinated fibers: 357 µm; percentage of muscle fiber area: 62.2%	
PCL-chitosan–gelatin	Model: In vivo Damage length: 15 mm Period: 12 weeks	Random	SFI: -59.23; CMAP: 10.76 mV; NCV: 36.2 m/s; myelin sheath: 0.68 µm; axon diameter: 2.25 µm; muscle weight ratio: 0.55	[55]
		Aligned	SFI: -52.31; CMAP: 13.66 mV; NCV: 46.0 m/s; myelin sheath: 1.3 µm; axon diameter: 3.17 µm; muscle weight ratio: 0.62	
PLLA	Model: In vivo Damage length: 5 mm Period: 8 weeks	Random	SFI: -71.1; count of axon: 76; myelin sheath: 0.51 µm; myelinated fibers diameter: 2.8 µm;	[56]
		Aligned	SFI: -59.9; count of axon: 102; myelin sheath: 0.62 µm; myelinated fibers diameter: 3.1 µm;	
GO-PCL	Model: In vitro Culture: 2D Period: 3 day	Random	Proliferation: 2.3 OD value; NGF expression: 1.7 pg/mL; poor spread: 102.6 µm;	[8]
		Aligned	Proliferation: 2.6 OD value; NGF expression: 2.1 pg/mL; poor spread: 301.9 µm;	
Silk nanofiber	Model: In vivo/In vitro Damage length: 10 mm Period: 12 weeks	Random	SFI: -74.31; NCV: 12.5 m/s; percentage of S-100 positive area: 11%; myelin sheath: 0.56 µm; diameter of myelinated axon: 2.7 µm; muscle wet weight ratio: 46.5%;	[57]
		Aligned	SFI: -66.3; NCV: 29.1 m/s; percentage of S-100 positive area: 29%; myelin sheath: 0.73 µm; diameter of myelinated axon: 3.3 µm; muscle wet weight ratio: 52.1%;	

*CMAP* Compound muscle action potential, *CNT* Carbon nanotube, *GO* Graphene oxide, *NCV* Nerve conduction velocity, *PCL* Polycaprolactone, *PLLA* Poly(L-lactide), *SFI* Sciatic functional index

**Fig. 2:**
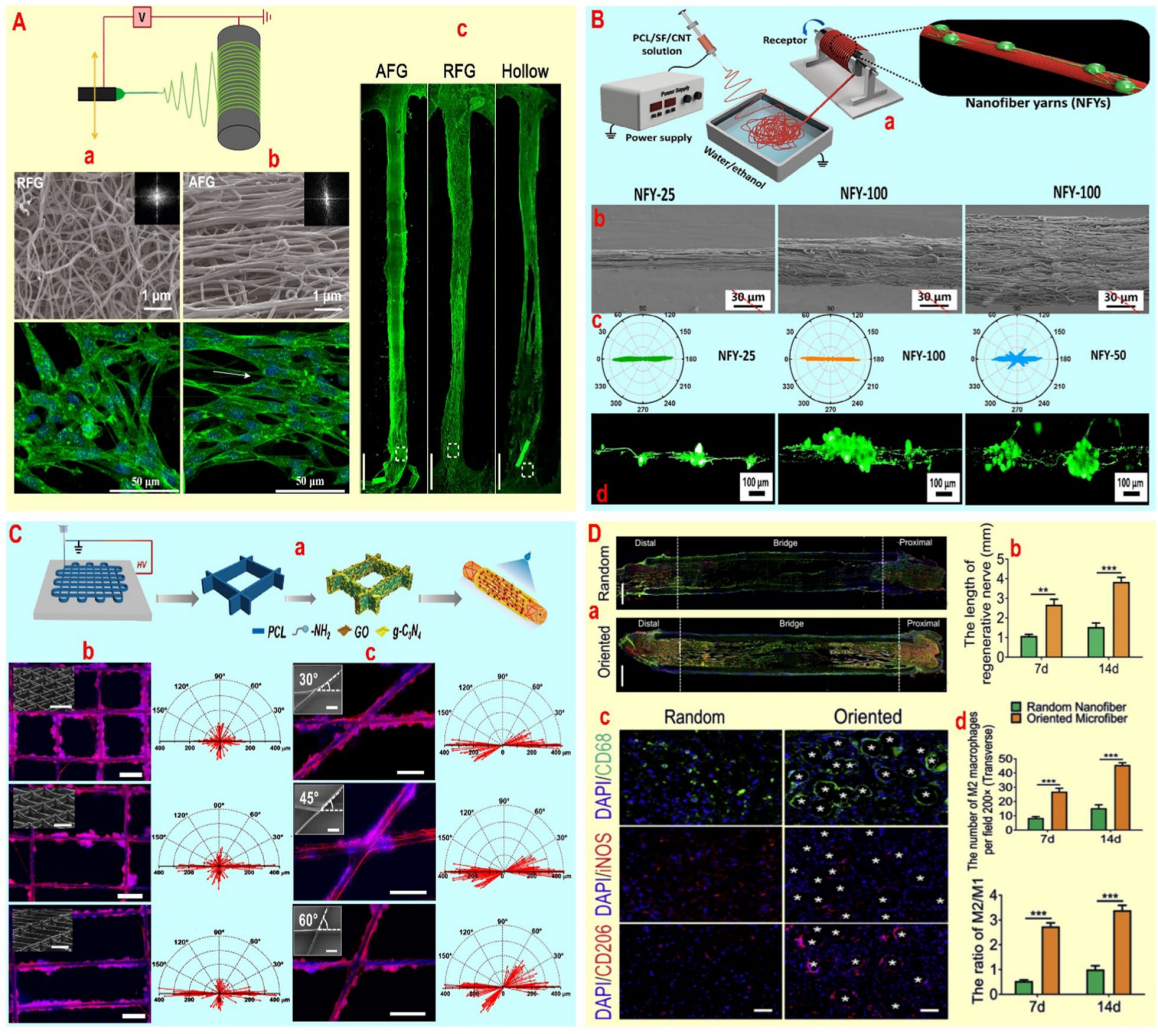
**A** SEM micrographs of AFG (a) and RFG (b), and their effects on nerve cell orientation. (c) Immunohistochemistry images of longitudinal sections of regenerated nerve tissues harvested from groups at 12 w after surgery. Axons were stained with NF200. Reprinted with permission from ref [54]. **B** Scheme of aligned NFY preparation by a dry–wet electrospinning method (a). SEM images of aligned NFYs with 25, 50 and 100 µm diameters (b). Fluorescence images of DRG cells on NFYs with varying diameters, stained with F-actin (green) and DAPI (blue) (c). Polar histograms showing neurite orientation distribution (d). Reprinted with permission from ref [63]. **C** Schematic view of conduit design decorated with photocatalyst (a). Immunofluorescence images of PCL cells in square and rectangular patterns (1–1, and 1–2 or 1–3) and corresponding plot (b). Immunofluorescence images of PC12 cells and corresponding plot of cell orientation (c) on parallelogram patterns, 30°, 45°, and 60°. Cells were stained with TUJ1 (red) and Hoechst (blue). Scale bars: 100μm. Reprinted with permission from ref. [64]. **D** Immunofluorescent staining of longitudinal sections, showing the distribution of neurofilaments (NF09, green) and Schwann cells (S100, red) in random and oriented nanofiber (a) and the length of regenerated nerve in conduits (b). Macrophage polarization characterized by CD68 (M0, green), iNOS (M1, red) and CD206 (M2, red) (c). The number of M2 macrophages and the M2/M1 ratio macrophages in conduits (d). Reprinted with permission from ref. [65]

Kim JI et al*.* [58] demonstrated that altering the conduit fibers from random to aligned within PLGA and PU (from electrospinning: voltage: 16 kV, flow rate: 1 mL/h) conduits containing NGF enhances the orientation of PC12 cells, aiming to improve nerve cell function. Aligned fibers were found to significantly improve the directional growth of PC12 cells by tripling the length and volume of intracellular actins parallel to the fibers. This enhancement was attributed to the reinforcement of cell adhesion, resulting from increased hydrophilicity that altered the cell contact angle from 128.3 to 120.2°. The alteration of PC12 cell morphology from flat or spherical in random fibers to spindle-shaped and oriented with aligned fibers validates this [58]. In next study, Chen S et al*.* [59] demonstrated that applying decellularized peripheral nerve matrix gel (pDNM gel) on PLLA fibers (diameter: 650 ± 90 nm) not only enhances axon growth by 30% through increased adhesion and interaction between DRG cells and fibers, but also leads to a 3–sixfold rise in Schwann cells migration in aligned fibers, significantly impacting the orientation of DRG cells. Schwann cell migration on aligned fibers and secretion of biological signals like NGF or neurotrophin-3 on them, have influenced the directional growth of neurons and axons extension. Moreover, it was demonstrated that the growth orientation of DRG cells significantly depends on the substrate pattern. It was observed that augmenting the thickness of the pDNM gel coating (from 0.25 to 1%) and decreasing the resolution of the substrate pattern lead to a more random growth of DRG cells. Surprisingly, the utilization of pDNM gel led to the emergence of fascicle-like axonal bundling in neural tissue, likely due to the increased presence of neuronal ECM molecules [59]. To study the impact of fiber alignment on nerve tissue function, Du J et al*.* [54] fabricated chitosan channels with aligned fibrin nanofibers using electrospinning (flow rate: 3 mL/h and voltage: 5 kV) and self-assembly. This led to increased DRG proliferation, adhesion, and directional growth of neurites/axons at 12 weeks post-surgery in a sciatic nerve transection in proximal region (10 mm) (Fig. 2A), along with increased NCV and CMAP (from ~ 5 to ~ 6.5 mV), neuron length, myelin sheath thickness (up to 30%), SFI (from − 60 to − 45), and gastrocnemius muscle-wet weight ratio (from ~ 0.41 to ~ 0.58%) [54]. Aligned fibrin nanofibers, similar to native tissue, facilitate the directional growth of neurites/axons and nerve cell orientation in under 2 weeks by enhancing cell migration and cell sprouting rate (0.65 mm/day). Nevertheless, the non-uniform and quick breakdown of fibrin over a 2 week timeframe presents obstacles for the targeted migration of neural cells in long-term healing. Quan Q et al*.* [60] used the PCL-chitosan conduit obtained from the electrospinning method (flow rate: 1.5 mL/h, voltage: 15 kV) to encourage the directional longitudinal growth of neurites/axons, without causing any toxicity to Schwann cells, PC12, and DRG despite the greater stability of the fibers. The poor adhesion of nerve cells on PCL polymer was effectively enhanced by utilizing chitosan. The notable enhancement in SFI recovery, gastrocnemius muscle mass, myelination, and distal nerve ultrastructure after 12 weeks of surgery, along with increased expression of ATF3 and cleaved caspase-3 after 7 weeks in the distal segment of transected sciatic nerve (15 mm), indicates the favorable alignment of fibers within the conduits [60]. Despite axonal protection and inducing directional growth in conduits with aligned fibers, repairing nerve defects above 2.5 cm remains challenging due to the uneven distribution of nerve cells along the conduit and the infiltration of connective tissues [61]. For this purpose, Dong X et al*.* [62] fabricated 30 mm composite porous conduits using PLCL in the shell and PDS in the center. They used electrospinning with a voltage of 16 kV and a flow rate of 2.0 ml/h for the shell, and electroprinting with a printing speed of 1800 mm/min and a voltage of 3.3 kV for the aligned fibers in the center. By seeding RSC96 and PC12 cells on microfibers, they successfully attained directional growth of neurites/axons and cells orientation with 0–10° alignment angle. The morphological results of RSC96 and PC12 cells suggest that composite conduits with aligned fibers prompt cells to elongate, exhibit a bipolar distribution of lamellipodia along the fibers, and enhance the development of Bungner structures in Schwann cell populations, which is vital for myelin formation. Moreover, the 30 mm severed dog sciatic nerve model demonstrated that the composite porous conduit enhances the physical structure of the sciatic nerve (diameter and flexibility), boosts blood vessel presence, particularly in the central area, and enhances nerve cell function. Furthermore, it was observed that the conduit containing aligned fibers inhibited the growth of invasive connective tissue within the conduit when compared to the control groups over a one-year period. Continuous and uniform distribution of the myelin sheath, optimal thickness, and density of myelin, increased axon diameter, higher NCV and CMAP, and improved gastrocnemius muscle structure confirm the positive impact of the conduit on enhancing the motor function of dog [62]. The experimental reports above indicate that 10–30 mm conduits with aligned fibers promote nerve regeneration more effectively than random fibers, enhancing cell migration and facilitating continuous tissue regeneration. Nevertheless, they do not address how structural aspects like aligned nanofiber thickness or junction shape impact cell functions and behaviors. Wang L et al*.* [63] demonstrated that the directional growth of neurites/axons and the orientation of PC12 or DRG depend on the diameter of aligned nanofibers. They found that increasing the fiber diameter from 25 to 50 μm expanded the rate of oriented neurites/axons from 62 to 64%, based on the angle between the long axis of neurites/axons with PCL-SF-CNT nanofibers coated with GelMA (Fig. 2B). Nonetheless, increasing the fiber diameter to over 50 μm significantly decreased the neuron orientation rate by 10%. The decrease in cell migration rate from 1380 to 1090 μm and the drop in cells sprouting rate from 460 to 360 μm per day due to the increase in fiber diameter from below 50 μm to above 50 μm were identified as the primary causes for reduced cell alignment. Expanding the surface platform without spatial limitations promotes the unrestricted growth of cells. In addition to fiber diameter, alterations in fiber connection angle and geometric pattern have been observed to affect nerve cells orientation. Decreasing the intersection angle from 90 to 30° and elongating the X-axis by changing the geometric pattern from square to rectangular increased the orientation rate of PC12 cells [64]. This implies that neurite elongation is equal in both the X and Y axes within square patterns. Changing the pattern from square to rectangle and increasing the length of the X-axis results in more PC12 cells being located on the X-axis (Fig. 2C). Additionally, altering the connection angle from 90 to 30° when transitioning the geometric pattern from a square to a rectangle leads to an exaggerated orientation of neurites along the X-axis [64]. Despite the increased orientation of neurites in anisotropic patterns with rectangular templates in vitro, the structural collapse caused by reduced mechanical strength continues to be a significant challenge.

Considering the primary and secondary inflammatory response is another key priority for enhancing PNI in the utilization of fiber-based support structures. In this field, using PLCL conduits coated with aligned PDS fibers (27.1 ± 3.9 μm in diameter) obtained by electrospinning (flow rate: 2 mL/h, voltage: 16 kV) and melt spinning (flow rate: 1 mL/h, temperature: 130 °C) methods, Dong X et al*.* [65] found that promoted PNI regeneration by attracting macrophages and polarizing them to an anti-inflammatory phenotype (Fig. 2D). In addition to improving migration, adhesion, and directional growth of DRG and Schwann cells, results show that aligned fibers supports nerve regeneration by reducing expression of TNF-α, IL-6, and iNOS genes, increasing oxidative phosphorylation, and promoting the polarization of M1 to M2 macrophages. Furthermore, the morphological change of macrophages to a quiescent state, higher CD206 levels, and greater presence of FIZZ1, IL-10, IL-13, and YM1 genes on microfibers confirm the anti-inflammatory characteristics of aligned fiber within conduits. Likewise, the findings from the transected sciatic nerve (11 mm) regeneration in middle segment demonstrate that alighned fibers vs. random fibers not only have a beneficial impact on neurites/axons length (3.9 vs. 1.5 mm) and DRG-Schwann cells orientation, but also indicate an improvement in the healing process within 14 days through increased the thickness and number of myelin sheaths (~ 200 vs. ~ 95), CMAP amplitudes (~ 65% vs. ~ 25%), and SFI (− 40 vs. − 85) [65]. Subsequent results revealed that in the transplanted tissue, macrophages and Schwann cells were aligned parallel to the microfibers. The close correlation between macrophages and Schwann cells indicates a strong impact of alignment on them, highlighting the significant influence of alignment on these cells. However, macrophages, especially M2-type macrophages, have a faster effect on nerve regeneration as neural progenitor cell guides through their faster migration on fibers compared to Schwann cells [65]. Vascularization of the regenerating nerve tissue is crucial, as is the control of inflammation for the stability and orientation of nerve cells on aligned fibers. Blood vessels seem to promote the healing process by providing a pathway for Schwann cell migration and supporting axonal regeneration through Bungner’s bands. Muangsanit P et al*.* [66] found that culturing HUVEC-Shawnn cells (4–0.5 × 10^6^ cells/mL) in silicone conduits with aligned collagen promoted sciatic nerve regeneration by increasing the number and length of vascular branches (based on CD31). The mechanisms described include Schwann cells calling and an increase in the number of nerve branches near the vessel. However, significant differences were only observed at the proximal stump, and the reduced number of neurites and axon regeneration at the distal stump make this approach difficult to use [66].

Neural cell migration, as described in Sect. 2.2, is a complex process facilitated by adhesion initiation and cell polarization through actin polymerization and microtubule organization. The aforementioned studies suggest that aligned fibers, similar to the native ECM, enhance focal adhesion and cytoskeletal rearrangement, such as actin, resulting in increased sprouting and nerve cones parallel to the aligned fibers and their expansion. Despite the uncertainty surrounding the mechanism of action of aligned fibers on migration and cell orientation, the theory of geometric potential influencing water expansion can elucidate part of the process. It has been observed that water spreads more effectively on aligned fibers compared to random fibers due to capillary properties [67]. Therefore, the increase in cell contact surface area resulting from higher hydrophilicity of fibers in aligned patterns enhances adhesion and cell migration, regardless of fiber diameter [63] and connetion shape [64]. A key mechanism involves enhanced interaction of pioneer cells with aligned fibers, leading to collective migration of neurons akin to tissue repair patterns. Utilizing leader cells with superior adhesive strength and migration speed, such as macrophages [65], can expedite and improve nerve cell orientation and regeneration. However, careful consideration of cell interactions and types when using leader cells alongside nerve cells is crucial to avoid challenges like fibroblast-induced scarring.

### Micro- or nano-patterns

The topology with repeated arrangements in natural neural tissue influences neuronal cell function, such as cell expansion and polarity, cell geometry, and signal transduction. So, meeting the topological requirements is essential to achieve the normal function of artificial neural tissue similar to natural tissue. The primary topological structures utilized for repairing peripheral nerves include grooves, channels, pillars and pits (Table 3).

**Table 3:** A summary of the pros and cons of methods for creating micro- or nano-patterns to regenerate nerve tissue [1, 9, 37, 68]

Structure	Manufacturing method	Benefits	Drawbacks
Fiber	Extrusion, phase separation, drawing, spinning (solution, electro, centrifuge, melting, gel, wet/dry), texturing, molding, printing	Similar to ECM in structure, wide range of materials, inducing cell orientation and directional growth of neurite/axons with fiber alignment, easy modification of fiber diameter, low cost, high porosity, easy implementation, diverse designs, simple surface modification, and quick access of cells to loaded materials	In aligned fibers: low mechanical strength, time-consuming production, increased structural collapse, increased cell migration, low cell adhesion In random fibers: low cell infiltration, calling of M1 type macrophages, low cell affinity, uncontrollable morphology and cavities
Channel	Lithography, printing, molding, freeze drying, solvent leaching, thermal-induced phase separation, electrospinning	High cell migration to depth, high cell orientation and directional growth of neurites/axons, controlling the invasion of adjacent tissues, reducing scars, simulating fascicles, controlling neuroma or nerve coils, high loading of neurotrophic factors, increasing the connection of two nerve ends	Reducing the penetration of signals and vital environmental secretions, unusual blockage of channels, complex production, reducing the diameter of fibers, lack of structural connection and signal between adjacent fibers
Groove	Lithography, molding, laser ablation, solvent casting, ion etching, electrospinning,	Mimic native tissues, high cell migration to depth, high cell orientation, manipulation of directional growth of neurite/axon, strengthening the high adhesion of cells, High variability of shape and size of grooves/edges in one platform	Complex production, accumulation of dirt/dust in between the groove, limited control on surface chemistry, stamp distortion, cell response dependent on groove/ridge size
Pillar	Lithography, molding, electron beam and hot embossing	High ability in promoting neurite growth, precise control over pillar placement, manipulation of neurite growth angles through pillar shape modification, enhanced cell adhesion, simulation of 3D culture, minimized cell death through optimal nutrient and waste flow	Complex design and long-time production, high costs, limitations on pillar height, resource constraints, poor substrate recovery, limited control over neurite growth location, reduced cell migration capacity, and poor cell orientation
Pit	Molding and solvent casting	Simple production, wide range of pit shapes and sizes, high flow rate of biological materials, extensive storage of compounds and medications, reinforcement of adhesion surface particularly in non-spherical edges, high flexibility	The risk of solvents, blockage of some pits, low cell orientation, poor directional growth of neurites/axons, presence of mega-pits

#### Grooves

**Fig. 3:**
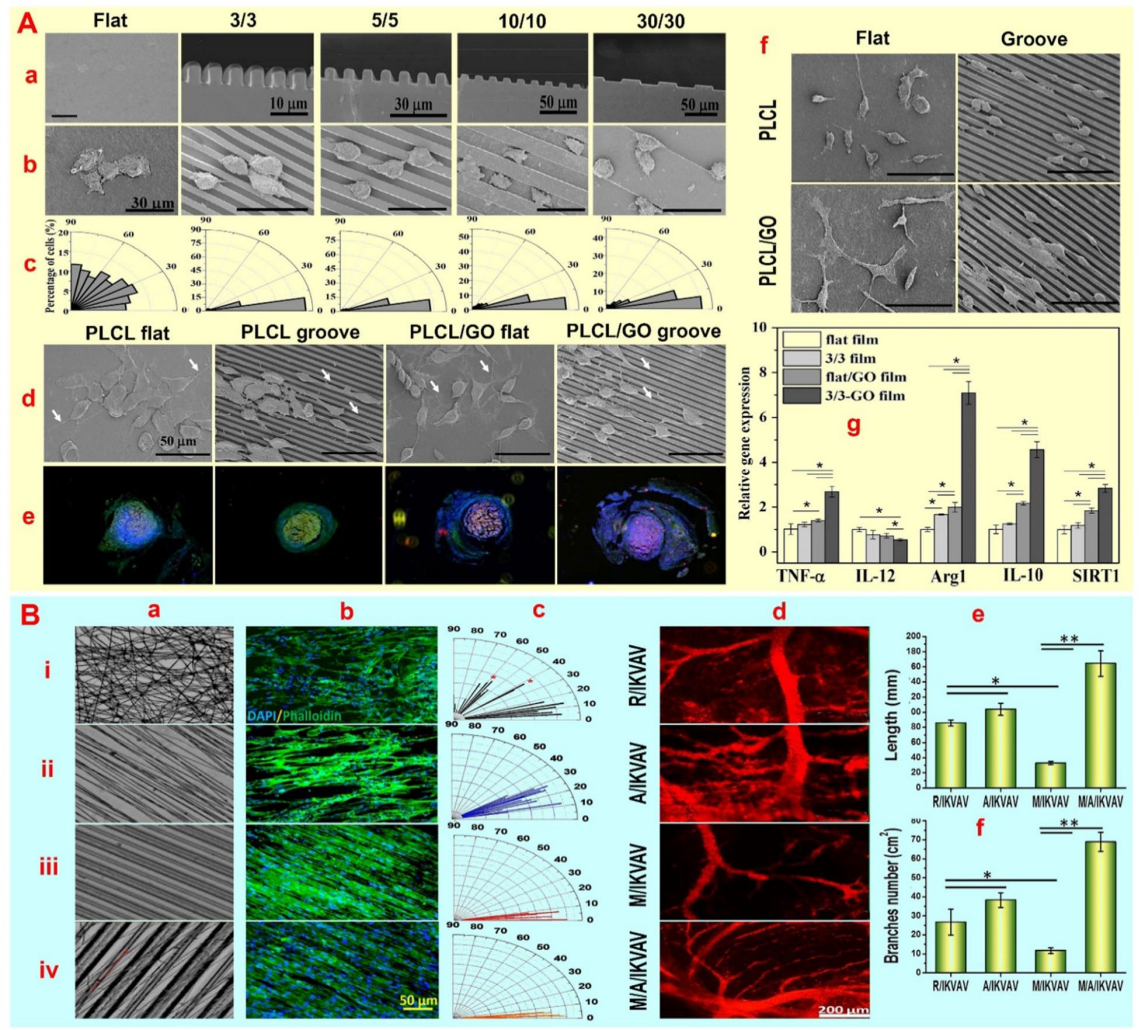
**A** SEM images of micropatterned films with ridges/grooves of 3/3, 5/5, 10/10 and 30/30 μm (a) and migration of Schwann cells on them (b). Distribution of cell orientation angle in films: flat, 3/3, 5/5, 10/10, and 30/30 μm (c). SEM images of neurite outgrowth (d), immunofluorescence staining images by S100β (SCs), NF200 (axons), and DAPI (cell nuclei) in the regenerated nerves (e), macrophage polarization (f) and relative gene expression of macrophages (g) on flat, 3/3, flat/GO, and 3/3-GO films. Reprinted with permission from ref. [76]. **B** SEM images of the random PCL fibers (R) (i), aligned PCL fibers (A) (ii), micropatterned PCL membrane (M) (iii), and micro/nanocomposite aligned topology (M/A) (iv) (a) and immunofluorescence staining of Schwann cells on them (b). Cell orientation plot on different platforms (c). Vascularization evaluation on various topographies coated with IKVAV peptide based on immunofluorescence staining using CD31 antibody for analyzing newborn blood vessels. Total length (e) and branches (f) of blood vessels in different groups. Reprinted with permission from ref. [77]

Changing the surface topography from smooth to rough always modifies nerve cell behavior (Table 4). Several studies indicate that groove/ridge patterns, resembling normal nerve tissue, play a crucial role in focal adhesion alterations and enhanced integrin clusters along the edges of the neural cell plane via the MAPK/ERK pathway [69, 70]. However, the molecular pathways influencing cell behavior alter with variations in depth and groove spacing. For instance, decreasing the ridge size from 1 to 15 nm with constant groove width changes the mechanotransduction pathway from MEK pathway to Rho-YAP pathway [70]. However, despite recognizing the impact of these patterns on nerve cell behavior, the optimal distance and depth of the groove/ridge on the nerve cell remain unclear. Increasing the width of these patterns appears to alter cell orientation by decreasing focal adhesions due to reduced nerve cell contact at the edges, while excessively reducing the groove/ridge distance leads to radial expansion in cell orientation. The bio-molecular mechanism induced by anisotropic geometries that change cell fate and induce directional growth is still not well understood due to limited access to geometric variations. Cai L et al*.* [71] showed that Schwann cell precursor line (SpL201) with glial nature and PC12 cells in 5 μm width and 12 μm depth grooves display enhanced cell morphology and more directional growth of neurites/axons. The alignment of cytoskeleton and cell nucleus with the grooves is improved by increasing groove depth (from 1 to 12 µm) and decreasing groove width (from 90 to 5 µm), while the cellular response to surface roughness is also affected by the nature of the cells and their motility. That is SpL201 exhibits less cell orientation compared to PC12 when the groove depth is changed from 12 to 1 μm. The higher adhesion of SpL201 cells seems to hinder cell migration than PC12, regardless of groove depth, due to increased integrin expression and enhanced focal adhesion maturation on the platform. Increasing groove depth by reinforcing the actin-myosin complex and Ca^2+^ influx from the membrane promotes cell migration in PC12 and SpL201 cells. While the reduction of the groove width through nucleus shape change affects microtubule organization and Ca^2+^ influx, it plays a role in cell orientation and migration. Therefore, the ideal spacing of aligned grooves for directing neurites/axons is determined by their depth. While it has been demonstrated that the width of grooves and ridges have a greater impact on cell orientation and migration [72, 73]. It has also been found that while reducing the width of grooves from 2000 to 500 nm with a depth of 350 nm has a positive effect on enhancing the directional growth of nerve cells, reducing the length of neurites is challenging in this process [70]. In addition, Huang C et al*.* [74] showed that modifying the surface of cellulose acetate butyrate conduits obtained through electrospinning (Voltage: 20 kV, flow rate: 0.8 mL/h) from smooth to aligned grooves with a width of 200 nm enhances the orientation of nerve cells in the injured sciatic nerve (≥ 15 mm). The increase in NCV (~ 45 vs. ~ 41 m/s), CMAP (~ 9 vs. ~ 7.5 mV), SFI and the wet weight ratio of gastrocnemius muscle (~ 67 vs. ~ 62%), as well as the increase in the number and thickness of myelinated fibers, validate the enhanced performance of the grooved pattern over the smooth pattern post 12 weeks of surgery [74]. In addition to directing nerve cell growth, the maturation of nerve cells during cell migration and orientation to enhance the repair process is a less explored area, as investigated by Polo Y et al*.* [75]. Aligned grooves (203.3 nm) with 496.5 nm ridges in the PLCL conduit had a positive impact on the directional growth of neurites/axons and cell orientation. However, graphene oxide or polydopamine coating on grooved surfaces led to greater maturation for neurons, as evidenced by increased neurite length. These coatings improved cell adhesion by increasing hydrophilicity and significantly enhanced cell orientation after neuronal cell differentiation. Nevertheless, compared to laminin, an ECM protein, the aforementioned coatings resulted in a slower rate of cell migration [75]. Similarly, Zhang D et al*.* [76] demonstrated that PLCL conduits produced using the cast approach in PTFE with graphene oxide coating exhibit enhanced cell adhesion and organization. They found that grooves/ridges measuring 3/3 μm on conduit surfaces, as opposed to other sizes (5/5, 10/10, and 30/30 μm), play a more significant role in cell migration, directional growth of neurites/axons (aligned with the grooves), and cell orientation after maturation (Fig. 3A). The improved adhesion of Schwann cells to graphene oxide nanosheets is attributed to the increased contact area and enhanced cell-substrate interaction by inducing NCAM and NCAD secretions (Fig. 3A). While, cell nuclei elongation and cytoskeleton formation along ridges/grooves, showing cell migration, result in a more accurate migration process that prevents random axon and neurite connections. Furthermore, this study shows that 3/3 μm aligned grooves/ridges coated with graphene oxide nanosheets can effectively promote the regeneration process by reducing inflammation. This is achieved by polarizing M1-type spherical macrophages into M2-type spindle-shaped macrophages through increased Arg1, IL-10, and SIRT1 expression (Fig. 3A). The improvement of SFI, CMAP, NCV, gastrocnemius muscle weight, the increase in myelinated axons, and the thickening of myelin all indicate the high effectiveness of 3.3 µm grooves/ridges with graphene oxide in regenerating transected sciatic nerve (10 mm) at 8 weeks post-operation [76]. The stability of the repair process relies on significant nerve cell proliferation, adhesion, and timely migration, while prioritizing angiogenesis is also crucial. Introducing biological markers consistent with the ECM structure of nerve cells, like laminin [75], can promote the angiogenic approach along with the rapid targeted distribution and adhesion of nerve cells. For instance, it was found that using IKVAV peptide markers on PCL aligned grooved surfaces (grooves/ridges: 11/9 μm) created by electrospinning (Voltage: 18.8 kV, flow rate: 0.088 mL/hour) improves the formation of multiple branches of blood vessels with diameter ≥ 50 µm aligned with the surface grooves and facilitated the directional growth of neurons/axons (Fig. 3B) [77]. Also, it was found that PCL aligned grooved coated with IKVAV peptide not only improved the proliferation and migration of Schwann cells based on the apparent distribution, but also markedly altered the cell orientation behavior from 0 to 90° (random fibers) and 0 to 35° (PCL aligned) to 0 to 10°. Meanwhile, it was observed that the development and expansion of blood vessels in aligned grooved groups lead to a higher degree of neuronal cell orientation and wider migration.

**Table 4:** Summary of the impact of micro-patterns on peripheral nerve organization

Type	Materials (M) Physical properties (P)	Method/Outcomes	Ref
Groove	M: PLCL-GN P: 20 µm size and 20.96 ± 1.26 MPa tensile strength	Model: In vitro/In vivo, Period: 12 weeks, Damage length: 10 mm, Outcome: Promoted cell migration, adhesion, and elongation, and the directional growth of neurites/axons. Its induce the myelin sheath, faster nerve regeneration and a 20-fold functional recovery	[78]
	M: CS-AS P: 46/18 μm groove/ridge sizes	Model: In vitro, Period: 3 days, Outcome: Increasing axon length by 80% and neurite length by 20%, reducing axon winding and promoting directional growth of neurites/axons, and enhancing cell orientation	[79]
	M: Silk fibroin P: 10, 30, and 50 µm size	Model: In vitro, Period: 3 days, Outcome: Enhancing cell orientation and directional growth of neurites/axons within a width of 30 µm, regulating cone growth along grooves	[80]
	M: PCL-PLA P: Sharp and wide ridges with 5 µm depth and groove of 15–20 µm	Model: In vitro/In vivo, Period: 16 weeks, Damage length: 10 mm, Outcome: Enhanced adhesion in wide ridges, increased directional growth of neurites/axons and cell orientation in short ridges, increased axon numbers, and cross-sectional area of axon in wide ridges	[81]
	M: CS P: Shapes of grating and isosceles triangle with 4 µm ridges, 6 µm groove, ~ 1.6 µm depth	Model: In vitro, Period: 3 days, Outcome: Despite the equal distribution among all platforms, the grating increased the directional growth of neurites/axons and cell orientation. It also reduced actin dispersion and increased cell polarization compared to other groups. However, cells on the scalene triangle platform exhibited the most displacement	[82]
	M: Gelatin-coated PLCL P: Groove/ridge/depth: 3/3/4 µm or 10/10/4 µm	Model: In vitro, Period: 24 h, Outcome: Grooves 3/3/4 μm had a greater impact than other groups, leading to increased migration, cell orientation, directional growth of neurites/axons, vinculin expression and adhesion by enhancing β1 integrin, Rac1, RhoA, and Cdc42	[83]
	M: PLLA-MTMC P: Groove/ridge/depth: 40/20/10 µm or 20/20/10 µm	Model: In vitro, Period: 5 days, Outcome: The 40/20 µm groove enhanced adhesion by cadherin, neurocan, and vinculin, leading to increased directional growth of neurites/axons, improved cell orientation, and well-organized filopodia and lamillopodia	[84]
Channel	M: PLATMC P: 4 channels: dimensions from 400 to 1400 nm	Model: In vitro/In vivo, Period: 12 weeks, Damage length: 10 mm, Outcome: Improved directional growth of neurites/axons, axon length, cell orientation, NCV (up to ~ 15%), and DCMAP (up to ~ 40%), coupled with enhanced cell number (~ 61%), myelin thickness (~ 50%) and vessels within the channels (~ 25%)	[85]
	M: Silk fibroin P: 5 channels: dimension ~ 570 µm	Model: In vitro/In vivo, Period: 12 weeks, Damage length: 20 mm, Outcome: By enhancing neurite/axon directional growth and improving cell orientation, the channels facilitated rapid recovery of the damaged tissue by increasing the number of nerve fibers	[86]
	M: Chitosan/N-succinyl-chitosan P: Conduit with internal fibers and holes of 66 µm	Model: In vitro/In vivo, Period: 12 weeks, Damage length: 10 mm, Outcome: Enhanced cell migration, directional neurites/axons length, myelin membrane, SFI, NCV, and gastrocnemius muscle, as well as improved cell adhesion and nerve cell viability	[87]
	M: gelatin methacrylate P: 4 channels with internal diameter of 1.2–2 mm	Model: In vitro, Period: 7 days, Outcome: Cell migration increased, especially in larger diameter, enhanced directional growth of neurites/axons, improved cell orientation, increased nerve buds, and enhanced cell junctions with each other	[88]
Pillar	M: PMMA P: Cylindrical shape, 6.5 µm pitch, 1 µm height	Model: In vitro, Period: 7 days, Outcome: Improving cell differentiation, increasing the length of axons and directional growth of neurites and axons	[89]
	M: PDMS@PEDOT:PSS P: 2.9–3.1 µm height and distance of 15 or 30 µm	Model: In vitro, Period: 24 h, Outcome: Increased adhesion, increased directional growth of neurites/axons, increased cell differentiation and enhanced cell orientation	[90]
	M: SU-8 P: 812–853 nm height, 430 nm diameter and distance of 1.2–1.22 µm	Model: In vitro, Period: 3 days, Outcome: Despite cell orientation and improved filopodia, pillars hinder the directional growth of neurites/axons, cell soma, and the differentiation of nerve cells. The combination of pillars with grooves further amplified these effects	[91]
	M: Silicon P: 5 µm height, 400 nm diameter and 0.8–5 µm distance	Model: In vitro, Period: 24 h, Outcome: Enhancing cell polarization, aligning neurites, and strengthening cell orientation to improve directional growth of neurites/axons and increase filopodia at 2 µm intervals	[92]
Pit	M: PCL, PLA, PCL-PLA P: the surface size ranged from 0.5–30 µm^2^ with 0.8 to 6.2 µm in diameter	Model: In vitro, Period: 6 days, Outcome: Despite the increased proliferation and differentiation of cells in the PCL and PCL + PLA groups, the directional growth of neurites/axons and the length of neurites showed greater increases in the PLA and PCL + PLA groups	[93]

*CS-AS* Chitosan-Artemisia sphaerocephala, *DCMAP* Distal compound muscle action potential, *NCV* Nerve conduction velocity, *PCL-PLA* Polycaprolactone-poly(lactic acid), *PDMS* Polydimethylsiloxane, *PEDOT* PSS Poly(3,4-ethylenedioxythiophene)-poly(styrenesulfonate), *PLCL-GN* Poly(l-lactide-co-caprolactone)-graphene, *PLLA-MTMC* Poly(-L-Lactide acid)- maleimide functionalized trimethylene carbonate, *PLATMC* Poly(lactide-co-trimethylene carbonate), *PMMA* Polymethylmethacrylate, SU-8 sulfonylurea

#### Channels

**Fig. 4:**
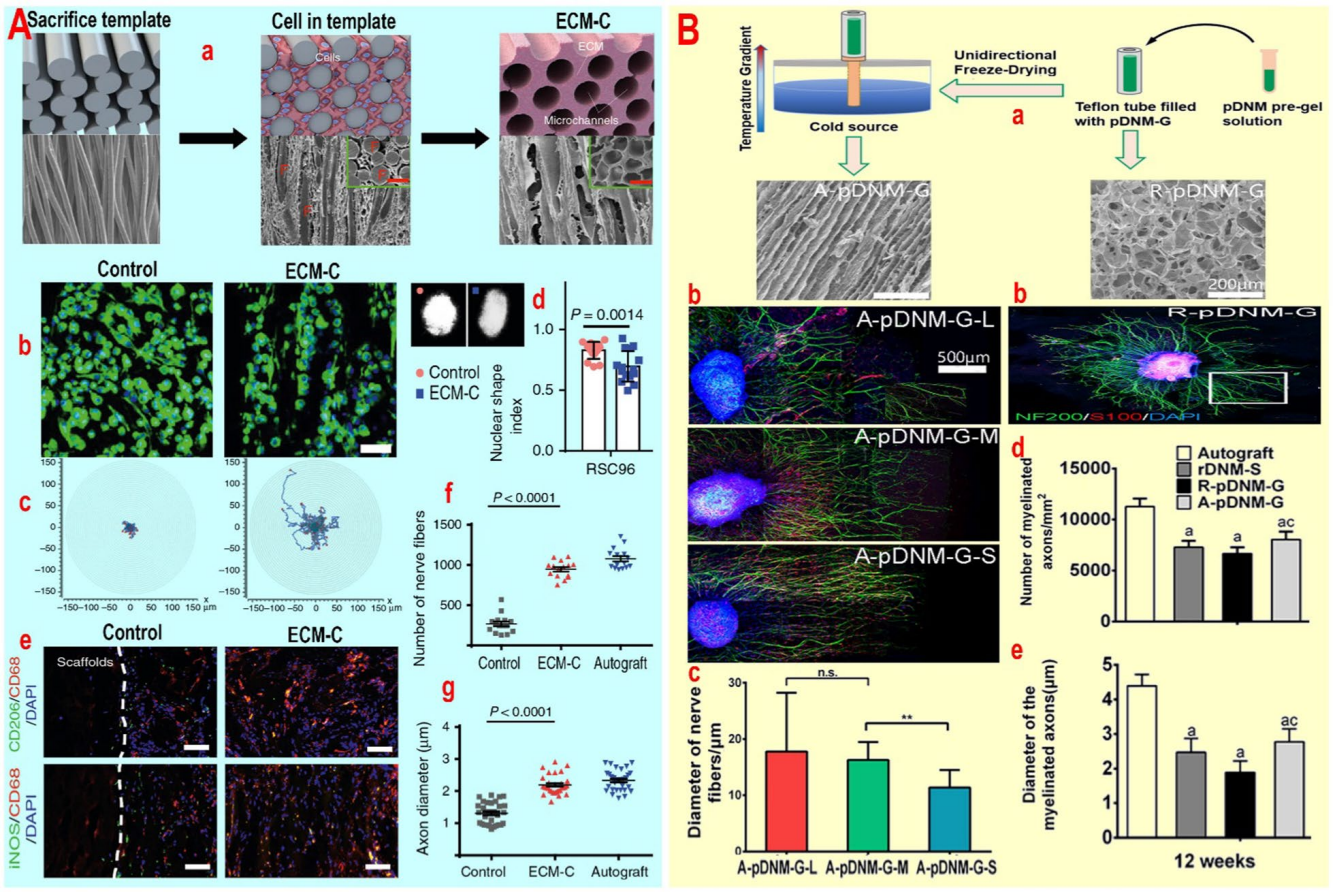
**A** The schematic diagram, the microstructure (SEM) of PCL fiber template, template-cell-ECM, and ECM-C during the preparation process (a). Inset: SEM examination of the transverse sections. RSC96 cells were stained with fluorescein isothiocyanate conjugated phalloidin and DAPI (b). Migration traces of RSC96 cells on different scaffolds (c). Typical images of RSC96 cell and the nuclear shape index of RSC96 cell on different scaffolds (d). Macrophages were detected by co-immunofluorescence staining for CD206 (green, M2)/CD68 (red, M0), and iNOS (green, M1)/CD68 (red, M0) (e). The number of myelinated fibers (f) and Axon diameter (g) among various scaffolds. Reprinted with permission from ref [94]. **B** Schematic illustration of the fabrication process of pDNM-G scaffolds and SEM imaging of R-pDNM-G and A-pDNM-G scaffold (a). Fluorescence micrographs of nerve cells on A-pDNM-G-L, A-pDNM-G-M, A-pDNM-G-S, and R-pDNM-G scaffolds, respectively (b). Quantitative analysis of the diameter of nerve fibers (c), the number of myelinated axons (d), and diameter of the myelinated axons (e). ^a^p < 0.05 between Autograft and the other groups. Reprinted with permission from ref [95]

Channel-type conduits are known for their ability to easily transport nutrients, load neurotrophic factors, and enhance cell migration. When compared to grooves, channels can more effectively prevent neuropathy and action potential challenge by isolating adjacent axons and neurons from extracellular fluid with low impedance. Also, based on the hypothesis of limiting three-dimensional growth space, channels increase the likelihood of reconnecting two nerve terminals by promoting the directional growth of neurites/axons (Table 4). However, despite numerous studies on the use of channel-type conduits in nerve tissue repair, challenges persist in regulating diameter, density, and alignment to the natural state. Decreasing channel diameter to prevent nerve coiling faces issues like channel blockage during physical activity or incomplete cell migration. Conversely, widening channel diameter increases the need for higher cell density, weakens nerve cell alignment, and may lead to nerve coiling. Thus, achieving an optimal diameter with high flexibility, degradability, and minimal inflammation remains a key research focus. For example, by designing SU-8 walled channels on a PDMS substrate and rolling them, Srinivasan A et al*.* [22] enhanced the controlled migration of Schwann cells, cell orientation, and directional growth of neurites/axons in the sciatic nerve. Although spatial dependence is crucial in promoting unidirectional growth and preferential migration of neurons, among the channels with dimensions of 50–150 µm, the 100 µm channel exhibited the most effective performance in sciatic nerve regeneration and, organized and directional growth. After 8 weeks of surgery, it was discovered that 100 μm channels significantly enhance nerve regeneration by reorganizing fascicles, particularly the epineurial layer in the entire regenerated nerve, leading to a full accumulation of axons and Schwann cells. This reconstruction nearly aligned with the distal end of the nerve, boosting the nerve tissue’s action potential while preventing neuroma and nerve entrapment. Furthermore, the presence of spontaneous and/or sensory evoked action potentials during ankle flexion/extension, pinching, and pad foot rubbing after 5 months demonstrated the beneficial impact of 100 μm channels on sciatic nerve regeneration [22]. In a separate study focusing on reducing inflammation, Zhu M et al*.* [94] developed collagen and elastin-rich channels (146.6 µm in diameter) by implanting aligned PCL (without inflammatory responses) sacrificial molds under rat skin (Fig. 4A). This method allowed for the orientation and directional growth of nerve cells along the channels, resembling normal tissue, despite the random culture of Schwann cells. The elongation of neural nuclei along the channels, the shift in cell migration pattern from radial to linear (Fig. 4A), and the increase in cell migration speed suggest that ECM-type channels actively contribute to the repair of neural tissue by promoting directional growth of nerve cells. However, the precise molecular mechanism underlying the channels' impact on the orientation and directional growth of nerve cells remains incompletely understood. In vivo studies on the sciatic nerve after 12 weeks showed that the ECM-type channel increases capillary quantity with structural and functional indices similar to normal capillary, enhances anti-inflammatory CD206-positive macrophages (M2), improves the density of oriented myelinated fibers, and increases axon diameter (Fig. 4A). These results suggest a significant impact of the channel on neural tissue regeneration [94]. Although the ECM has positive effects on neural tissue regeneration, the control of directional growth of neurites/axons and cell orientation seems to depend on the canal diameter and its topological structure. By creating aligned channels of varying diameters from decellularized porcine neural trunk hydrogels using a one-way freeze-drying method, Rao Z et al*.* [95] were able to significantly enhance the directional growth of neurites/axons and cell orientation by reducing the channel diameter from 96 ± 27 μm to 53 ± 13 μm or 29 ± 8 μm (Fig. 4B). The temperature gradient-induced reduction in channel diameter improved neurite growth from radial to linear patterns, resulting in a twofold increase in neurite length, but also led to an unintended decrease in fiber diameter (Fig. 4B). Increasing the channel diameter promotes the bundling of nerve fibers and supports neuronal development. Also, investigations of sciatic nerves severed in the distal region 12 weeks post-surgery revealed that the canals expedite healing by enhancing Schwann cells and DRG migration and infiltration, increasing myelinated axon count, myelin thickness (Fig. 4B), nerve fiber thickness, NCV, CMAP, SFI and the weight of the gastrocnemius muscle [95]. While ECM proteins like fibronectin or laminin have been found to positively impact cell adhesion and differentiation, Pawelec KM et al*.* [96] demonstrated that applying fibronectin or laminin coating to PCL or PLGA channels did not significantly affect the growth direction of axons/neurites or cell orientation. Conversely, creating aligned grooves (40 μm) on PDMS substrates within channels facilitated the directional growth of axons/neurites and the orientation of neurons. Additionally, it was found that co-culturing DRG with Schwann cells and adjusting channel wall porosity did not influence the directional growth of neurites/axons and cell orientation, although it did impact angiogenesis and neurites length [96]. In the above confirmation, it was found that SP neuropeptide coating increased neural stem cell calling and reduced inflammation without affecting the directional growth of PC12 [97]. Altering the structure of PLCL coated with SP neuropeptide from planar to channel by MEMS-based micro-patterning resulted in a decreased alignment angle between the long axis of the channel and the axis of the PC12 neurites in the transected sciatic nerve model.

#### Pillars

**Fig. 5:**
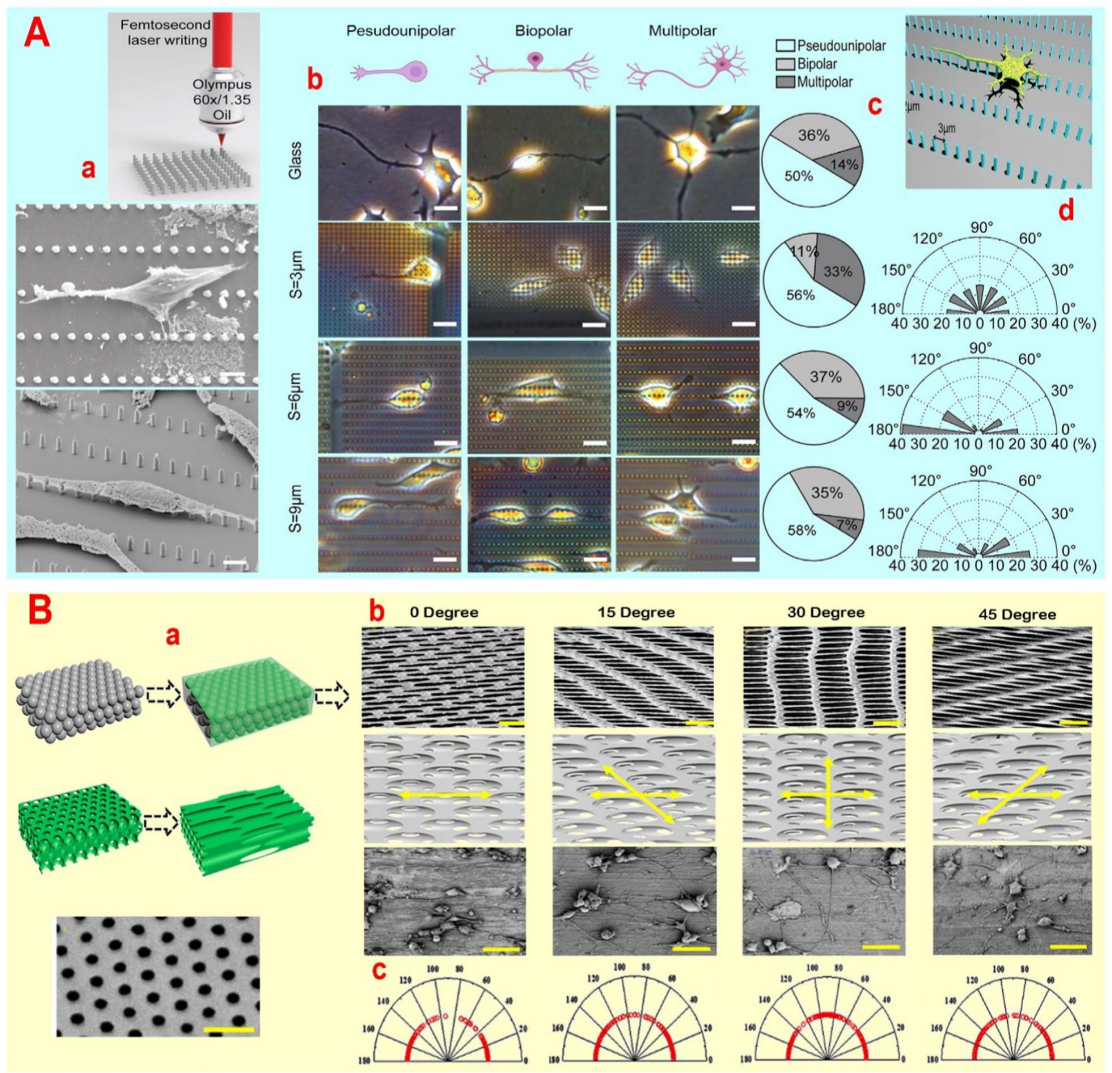
**A** Schematic diagram of fabrication and SEM images of neural stem cells cultured on anisotropic micropillar scaffolds with a distal distance of 6 µm guide (up) and 12 µm guide (down) neurite growth. Scale = 4 µm. Phase contrast figures show the nerve cell growth morphology at different groups (b). Scale = 20 µm. Pie charts show the percentage of neurite branches on glass substrates and micropillar scaffolds with spacing of 3, 6, and 9 µm (c). The orientation of neurites is represented by the orientation angle distribution (d). Reprinted with permission from ref. [101]. **B** Schematic view of scaffold construction (a). SEM images of the scaffolds stretched at different angles and scheme of tailored inverse films with different stretching directions (b). SEM imaging of cell culture on films stretched in different directions based on angles of 0°-, 15°-, 30°- and 45°-stretched (b) and angle distribution of neurites of PC12 cells (c). Reprinted with permission from ref [109]

Topographic cues typically impact development, differentiation, and directional growth by lessening surface constraints and offering contact guidance to promote cellular connectivity. Numerous studies indicate that columns, alongside other topographies, impact neuronal morphology (Table 4), resulting in enhanced elongation, preferential growth, and alterations in nuclear shape [21, 98]. However, the various responses regarding the impact of pillar distance and height on nerve cell orientation and growth, irrespective of pillar shape, necessitate further research. Regardless of the influence of pillar shape on the emergence of growth cones (for instance: increased preferred directions in the hexagonal shape compared square and circular shapes [99]), pillars (height: 3 μm) significantly increased the directional growth of neurites/axons through Golgi-centrosome accumulation (up to ~ 67%), N-cadherin amplification (62–67%) and actin higher accumulation [100]. Also, based on the F-actin probe, it was revealed that the first nerve cones are located near the pillar signal, irrespective of the pillar spacing. According to pillar density, it was found that the first signs of directional neurite/axon growth and a doubling of neurite/axon length are seen in pillars with distance of less than 2 µm. While, the highest average growth of cone area is noted at distances ranging from 2.4 to 5 µm. Consequently, it appears that an overabundance of nerve cell branching is hindered due to the limited number of growth cones and their constrained advancement at distances under 2 µm [100]. Despite the positive impact of pillars on actin accumulation and the formation of N-cadherin crescents to promote sprouting, the mechanism by which pillars influence cell polarization and the directional growth of neurites/axons remains unclear. Nevertheless, the filopodia of initial growth cones promptly come into contact with pillars upon sensing the environment, serving as anchoring points that determine the direction of growth, migration, and consistent growth rate. In the following, Repić T et al*.* [21] demonstrated that neonatal neurons showed a preference for sites with closely spaced pillars ranging from 0.6 to 1.4 μm, whereas adult neurons favored pillar spacing of 1.6 to 2.4 μm. The variation in integrin expression levels between adult and newborn neurons may be the cause of their differing expansion capabilities. They demonstrated that increasing the spacing of pillars to over 3 μm eliminated the topographic guidance effect for directional growth of neurites/axons and cell orientation. While, Fan S et al*.* [101] demonstrated that pillars (height: 6 μm; diameter: 1 μm) created by direct femtosecond laser writing technology in an SZ2080 photoresist platform, with a distance of more than 3 μm, can significantly impact the directional growth of neurites/axons (Fig. 5A). They noticed that increasing the distance of the pillars from 3 µm to 6 and 9 µm results in the directional growth of neurites/axons from the distal position (the distance between two pillars in different rows) to the proximal one (the distance between two pillars in a row) (Fig. 5A). Increasing the distance of pillars from 3 µm to 6 and 9 µm led to a decrease in multipolar neurites from 33 to 9% and 7%, and an increase in bipolar neurites from 11 to 37% and 35%, respectively [101]. Nevertheless, Vedaraman S et al*.* [102] demonstrated that increasing the distance between the wall-like pillars (length: 20 µm, width: 1 µm, height: 10 µm) by more than 20 µm in the anisometric microstructure coated with PLL inhibits cell orientation and the directional growth of neurites/axons. Despite the induction of cellular structures such as lamellipodia and cellular filopodia by wall-like pillars, it was found that beyond a certain threshold distance, only more mature and larger neurons were able to detect surface heterogeneity. Furthermore, it was noted that the orientation of neurites/axons is not solely determined by topography and is impacted by neighboring cells [102]. Chemical signals from cells and transient or persistent junctions seem to regulate this process. Moreover, this finding suggest that the impact of supporting cells can at times outweigh that of wall-like pillars, enabling axons to extend against the main axis.

Increasing the height of the pillars has been shown to change the directional growth pattern of neurites/axons and the preferential alignment of cells, in addition to the distance between the pillars. For instance, raising the height from 25–50 to 100 nm resulted in the growth cones and directional growth of neurites/axons aligning parallel to the pillars [103]. This is due to the increased adhesion of neurons to taller pillars. In a subsequent investigation of the SiO2 substrate coated with PLL, it was discovered that the development of actin cytoskeleton on ~ 400 nm pillars, with paxillin-rich regions, is notably greater than on the smooth surface [104]. Increasing the height of the pillars up to 400 nm, as opposed to 100 nm, restricts neurite growth by confining the available space and causes a biased alignment of neurites/axons with the topographical pattern. Furthermore, the distribution of neurites/axons on pillar arrays indicates that their arrangement on 400 nm pillars remains consistent with the expected pattern, as opposed to 100 nm pillars [104]. Recently, Milos F et al*.* [105] demonstrated that the combination of light-stimulation with light-sensitive P3HT pillars (height: 6.4 ± 0.3 μm, diameter: 2.3 ± 0.1 μm) coated with PLL improves the directional growth of neurites and axons at specific angles. Additionally, it was discovered that P3HT pillars boost the adhesion and growth of neurites/axons by 40% through the accumulation of paxillin in the side walls. Additionally, stimulating neurons on the platform with P3HT pillars resulted in a notable increase in neurite length compared to neutral substrates, without causing any toxicity to neurons [105]. While photo-stimulation has positive effects, the limited penetration of visible light, particularly in deep tissues, is a drawback in clinical practice.

#### Pits

Cell studies suggest that pits, along with other micro- and nano-patterns (Table 4), can create substantial focal adhesions when interacting with cells. The shape of the pits has a significant impact on the focal adhesions formed as a result of cell-pit interaction. For instance, square-shaped pits have been discovered to promote focal adhesions by causing actins to accumulate more at the corners of the pits, whereas round pits result in more uniform focal adhesions with less actin [106]. In this regard, Mobasseri S et al*.* [107] created a PCL/PLA film with pits using a solvent casting method. They demonstrated that the presence of pits enhances neurite adhesion on surfaces with pits as opposed to smooth surfaces. Nonetheless, pits showed limited effectiveness in promoting neurite/axon directionality and cell orientation when compared to grooves and pillars. Whereas, another study found that 200 nm pits in aligned PLLA fibers, which were coated with poly-pyrrole nanoparticles, resulted in a ~ 55% increase in cell orientation and an ~ 84% increase in directional growth of neurites/axons when compared to aligned fibers without pits [108]. Also, pits-containing fibers were found to improve adhesion for PC12 cells along the fibers by strengthening filopodia through actin accumulation and microtubule rearrangement, as opposed to smooth fibers [108]. The anisotropic poly(3,4-ethylenedioxythiophene): poly(styrenesulfonate) mixed polyacrylamide polymers fillers with interconnected pits demonstrated that morphological changes and rearrangement of pits during film stretching significantly impact the adhesion, orientation, and networking of expanding nerves (Fig. 5B) [109]. Neurons exhibit directional growth on these layers under different stretching conditions compared to glass slides. For instance, during 180° stretching, neurite growth and cell orientation align with the stretching horizon. Altering the angle from 180 to 15°, 30°, and 45° affects cell orientation based on the arrangement angles of the pits (Fig. 5B). It was observed that for optimal neural network formation, cells orient longitudinally during 180° stretching and at a 30° angle [109]. Recently Piscioneri A et al*.* [110] demonstrated that the alignment of pits in PLGA layers, achieved through lithography, significantly influences cell orientation and the directional growth of neurites/axons through spatial and temporal organization. However, the impact of pits on cell orientation is notably less significant compared to grooves and pillars.

## Synergism between support structures and metal nanoparticles

**Fig. 6:**
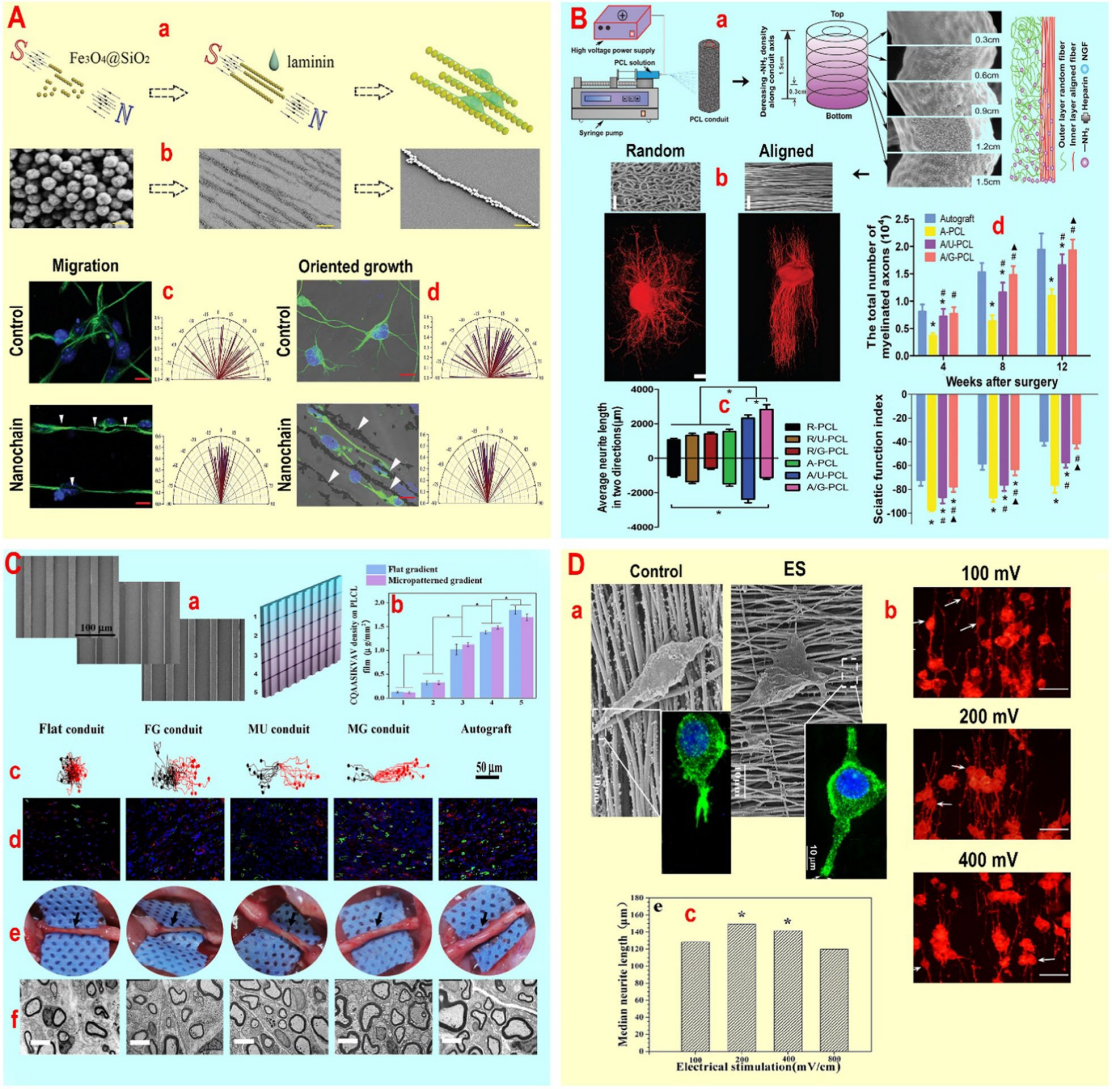
**A** Assembly of magnetic nanochains and guided oriented growth of the neurons (a). Fe_3_O_4_@SiO_2_ nanoparticles, single magnetic nanochain, and overview of magnetic nanochains observed by SEM (b). Representative fluorescent photographs of cells migration in groups (green: nestin; blue: DAPI), and orientation angle plots (c). Representative fluorescent photographs of directional growth of neurons in groups and Orientation angle plots (d). Reprinted with permission from ref. [116]. **B** Schematic diagram of NGF-gradient/aligned PCL conduits fabrication by electrospinning, and NH2-Heparin-NGF group gradient was formed by the dynamic reaction of ethylenediamine solution on PCL (a). Nerve cell neurite outgrowth and orientation on fiber morphology of R-PCL and A-PCL conduits (b). Average neurite length in two directions (c). Number of myelinated axons and sciatic functional index in conduit utilization (d). Reprinted with permission from ref [121]. (**C**) SEM images of micropatterned PLCL films after being aminolyzed for 2, 4 and 8 min (a). Scheme to show the five parts of gradient in micropatterned gradient PLCL and the peptide density on the surface of micropatterns (b). Migration traces of nerve cells (c), immunofluorescence staining images of CD163 (green), CD86 (red), and DAPI (blue) for M2, M1 and cell nuclei in regenerated nerves (d), optical images of regenerated nerves in vivo (e) and TEM images of nerves guided (f) by Flat, FG, MU, and MG conduits, and autografts, respectively. Reprinted with permission from ref [122]. **D** SEM images of PC12 cells on aligned PPy-PLLA fibers without and with electrostimulation of 200 mV/cm, and inset fluorescent images of PC12 cells with two growth cones (a). Fluorescence images of PC12 with electrostimulation of 100, 200, and 400, mV/cm, respectively (b). Median length of neurites from PC12 with under different electrostimulations (c). Reprinted with permission from ref [24]

As previously mentioned, micro and nano patterned surface topography enhances the nerve regeneration process by promoting cell adhesion, targeted migration, and directional growth of neurites/axons. However, variations in surface stiffness and transmission of signals within these patterns can cause the low reproducibility of cell orientation responses. The presence of metal nanoparticles like IONPs in supporting structures appears to influence cell behaviors consistently, similar to natural structural patterns in the body, enhancing cell orientation [111]. IONPs synchronize two hypotheses: (1) activating cell adhesion molecules via cell–matrix interactions by triggering the iron-related MAPK pathway, and (2) Orienting nerve cells by aligning iron nanoparticles attached to cells in a magnetic field. Indeed, nerve tissues intrinsically facilitate filopodia growth and cell orientation for directional growth of neurites/axons through the transduction of remodeling signals into supporting structures via metal IONP nanoparticles or Mn nanoparticles (e.g., RGD-dependent ECM for migration and differentiation) [112]. Meanwhile, the random patterns penetrate deeper into the nerve cell membrane and strengthens mechanical signal transmission [113]. However, the lack of directed growth of neurites/axons and non-targeted migration limits the use of random patterns. In this regard, Antman-Passig M and Shefi O [114] successfuly improved neurite/axon growth direction by aligning 100 nm IONPs under a 255G magnetic field in collagen hydrogel, despite the adverse impact on the number and length of neurite branches. The synergy of aligned collagen fibers with aligned IONPs resulted in a ≥ 60% correlation of cells with the aligned line, leading to enhanced neurite/axon directional growth up to twice compared to the control group. They demonstrated that it is possible to inject the gel with aligned strands of collagen and IONPs into the damaged environment without the requirement for pre-implantation treatment, by ensuring the stability of the alignment of IONPs in the collagen hydrogel after remote treatment with a magnetic field. However, when IONPs were aligned in the non-aligned hydrogel, it led to a decrease in the alignment and length of neurites/axons, even though aligned IONPs had a positive impact on the orientation of nerve cells. In next study, Antman‐Passig M et al*.* [115] showed that combination of IONPs with aligned collagen fibers increased DRG cell orientation by 13% (from 39 to 52% at ± 15°) compared to aligned collagen fibers alone. While it was discovered that the synergism of NGF-coated IONPs (11.3 nm) with aligned collagen fibers did not significantly affect the orientation of DRG cells. Nonetheless, this synergy notably enhanced the length of axons and neurites when compared to separate methods. The effects of IONPs were more pronounced compared to NGF on neurite length, directional growth, and cell orientation. During the repair of transected sciatic nerves (≥ 10 mm), it was discovered that the synergism of NGF-coated IONPs compared with IONPs and NGF with aligned collagen fibers enhances axon count (5152 ± 908, 2737 ± 419, and 1723 ± 374, respectively) and promotes their directional growth. This effect is particularly notable in the middle and distal regions where axon growth is evenly distributed. The enhanced rat locomotor performance, as indicated by increased SFI and CMAP, further validates the beneficial synergistic impact of NGF-coated IONPs with aligned collagen fibers [115]. Xia L et al*.* [116] recently achieved a significant increase in the directional growth of neurites/axons and nerve cell orientation by synthesizing laminin (1%)-coated Fe_3_O_4_@SiO_2_ nanowires. Statistically, it was found that over 70% of neural stem cells and 50% of neurons derived from stem cells with an angle of less than ± 10° were significantly dispersed on the nanochain compared to the control group (12.7% of neural stem cells with a direction angle less than ± 10°) (Fig. 6A).

In the follwong, Rose JC et al*.* [117] designed 50 × 5 × 5 μm^3^ PEG microgels containing SPION in the anisogel system to create an aligned platform for the directional expansion of nerve cells using a magnetic field. They found that microgels alignment at an optimal distance of 33.6 μm from each other promotes the directional growth of neurites/axons and cell orientation. However, the optimal concentration of microgels for different cell and tissue types remains unclear. Because increasing microgels concentration from 0.5 to 1% in fibrin hydrogel positively affected neurite/axon growth direction, but concentrations over 1% did not show significant impact. While in a similar study, increasing PLGA microgels concentration containing SPION (4.2–6.2 nm) from 1 to 5% and 10% notably decreased cell orientation by reducing microgels alignment [118]. However, it seems that aligning microgels in fibrin hydrogel promotes the directional growth of neurites/axons by providing aligned platforms.

## Synergism between support structures and the biological agent gradients

One effective way to enhance nerve cell migration, cell orientation, and directional growth of neurites/axons is by utilizing biological gradients in support structures with micro- or nano-patterns. The use of soluble molecule gradients as chemotaxis to direct cells has been well-known for more than a century. However, the certainty of gradient loading concentration on templates, high reproducibility of targeted loading, and the diffusion state of gradients still face uncertainties. Nanotechnology seems to have offered new opportunities where chemotaxis by nano- and microcarriers, along with haptotaxis provided by substrate-bound molecules, can generate a concentration gradient. The combination of chemotaxis or haptotaxis with micro- or nano-patterns appears to improve the directional growth of neurites/axons and cell orientation. In this regard, Kundu A et al*.* [99] demonstrated that applying a Netrin-1 gradient on 3 μm high pillars coated with poly-L-lysine enhances the directional growth of neurites/axons and growth cones in DRG cells more effectively. While the ideal spacing between the pillars for guiding neurite growth was found to be 1.4 μm, applying Netrin-1 to the pillars within distances ranging from 0.6 to 5.6 μm could potentially adjust the optimal spacing to 1.8 μm. The synergy of the biological gradient and topographical cues enables the modification of neurite extension and speed. However, the intricate architecture of conduits with biological gradients poses a challenge for commercial utilization. To address this issue, Alsmadi NZ et al*.* [119] proposed a straightforward approach involving the creation of a multi-channel (250 μm) conduit by depositing 1.5% agarose on coiled PLGA polymers containing NGF. In this approach, polymer coils with NGF established a spectral gradient ranging from high (concentration of 48 ng/ml/day) to low (39.4 ng/ml/day) depending on the polymer's coils in the conduit, enhancing the guidance of neuron growth towards the distal side. They demonstrated that the synergy of NGF gradient with the channel resulted in a twofold increase in the directional growth of neurites/axons, PC12 or DRG cell orientation, and increased axon length (up to 60%) by reducing their rotation angle. Besides the beneficial impact of the gradient approach, altering their concentration by increasing the amount of PLGA coils also influences the longitudinal growth of axons (from ~ 55.76 mm in NGF less than 50 ng to ~ 87 mm in NGF above 50 ng) and their directional spread [119]. Similarly, NGF gradients induced by ionic junctions from Pluronic F127 + heparin in porous PCL conduits were found to enhance the repair rate of transected sciatic nerves (20 mm) from ~ 0.24 mm/day (bare conduit) and ~ 0.36 mm/day (NGF uniform group) to ~ 0.71 mm/day [23]. The acceleration in healing time from 12 and 8 weeks in bare conduit and NGF uniform group to 4 weeks in the NGF gradient group, respectively, highlights the beneficial impact of the collaboration between the NGF gradient and the porous PCL channel. The rapid improvement in rat motor performance with an increase in nerve fiber diameter, myelin sheath thickness, NCV, and weight of the gastrocnemius muscle validated the beneficial impact of NGF gradient and PCL channel synergy [23]. In this line, Hsu RS et al*.* [120] demonstrated that the synergy of NGF gradient with porous GelMA channels enhances schwann cell migration, elongation, axon growth, and directional growth of neurites/axons. The NGF release profile from the GelMA channel reveals a time-dependent release influenced by polymer erosion and degradation, accelerated by collagenase. Consequently, regulating NGF release from the polymer to establish a biological gradient faces challenges in vivo, particularly due to uneven polymer degradation in biological settings. Nonetheless, the enhancement of SFI (− 45 vs. − 66), NCV (~ 45 vs. ~ 30 m/s), myelin layers (~ 39.4 vs. ~ 22.1 number), and nerve fiber diameter (~ 4.7 vs. ~ 2.1 µm) post 60 days of transected sciatic nerve surgery (5 mm) signifies a positive effect of NGF gradient and GelMA channels compared to a bare GelMA channel. Based on the printing method and loading specific concentrations of NGF on the SF/Collagen conduit with 15.74 ± 37.82 µm aligned holes, Huang L et al*.* [7] successfully established the NGF gradient despite maintaining a consistent release rate of NGF along the conduit. This strategy led to a gradual reduction in neurites/axons growth as diffusion declined over time, while higher concentrations accumulating in certain regions stimulated directional neurites/axons growth. In the same way, it was found that combining NGF gradient with aligned porous resulted in aligning 81.3 ± 4.5% of neurites/axons at an angle of less than ± 10° and cell orientation, surpassing the alignment achieved by aligned porous alone (63.8 ± 3.7%). The enhanced longitudinal growth of axons, increased number of myelinated axons, larger fiber diameter and myelin thickness sheets, along with the improved SFI, demonstrate the beneficial impact of this synergy in the transected sciatic nerve model [7]. While 3D printing has shown success in creating conduits with gradient biomaterials, its application is hindered by high costs, production complexity, slow speed, and the incapacity to generate micro- and nano-patterns on a single platform. Therefore, Zhu L et al*.* [121] facilitated the NGF gradient more effectively by submerging a PCL conduit with aligned fibers (3.1–4.6 m) in an ethylenediamine solution containing a -NH_2_ gradient, and then linking NGF to them within the conduits (Fig. 6B). Synergy of NGF gradient with aligned PCL fibers significantly promoted directional growth of neurites/axons, cell migration, and cell orientation (Fig. 6B). Despite the positive outcomes of this synergistic impact on sciatic nerve regeneration in terms of enhancing SFI, NCV, myelin thickness, nerve fiber diameter, and gastrocnemius muscle weight, the potential toxicity of -NH_2_ post NGF release remains unexplored (Fig. 6B). However, it was discovered that the NGF gradient is detected by distal axons. This detection, through the stimulation of Adcy1, Prckz, Rap1/PI3K/AKT, and MAPK pathways, enhances axon growth and adhesion, guides growth cones, and promotes synaptogenesis on aligned PCL fibers [121]. Recently, Zhang D et al*.* [122] achieved enhanced migration rate, cell orientation, and directional growth of neurites/axons by developing a grooved PLCL platform (40 (groove)/20 (ridges) µm) and loading CQAASIKVAV peptide gradient using glutaraldehyde amino agents. The study demonstrated that the combination of grooves with a peptide gradient led to the formation of more organized filopodia and lamellipodia, a spindle-like cell morphology, and longer neurites when compared to other groups (Fig. 6C). Despite the potential toxicity of glutaraldehyde, no toxicity was detected in this system. Additionally, the peptide gradient was found to enhance synergistic activity, leading to improved polarization of M1 to M2 macrophages and angiogenesis (Fig. 6C). Furthermore, the synergism of grooves with the peptide gradient was observed to boost the regeneration rate of the transected sciatic nerve (Fig. 6C), resulting in larger fiber diameter, thicker myelin membrane, longer neurites, and increased SFI, NCV, and CMAP [122].

## Synergism between support structures and the electrical stimulation

Several studies confirm that electrical stimulation enhances nerve cell proliferation, differentiation, migration, and integration by activating ion channels and influencing growth patterns [123]. The electric field influences the directional growth of neurites/axons, while the electromagnetic field promotes neurogenesis [124]. Hence, the synergism of electrical stimulation with supporting structures is expected to enhance the directional growth of nerve cells and their incorporation into healthy tissue. For instance, it was found that aligned fibers (diameter: 800 nm) composed of PPy-coated PLLA, when subjected to electrical stimulation of 100, 200, and 400 mV/cm, increased neurite extension from 68% along the fiber axis to 76%, 83%, and 71%, respectively [24]. The highest directional growth rate of neurites/axons and cell orientation was observed with electrical stimulation of 200 mV/cm (Fig. 6D). Moreover, the results show increased directional growth of neurites/axons in random fibers with electrical stimulation, indicating a positive response of nerve cells along the electropotential direction (Fig. 6D). Furthermore, the rise in longitudinal fiber growth from 65.44 and 114.73 µm in random and aligned fiber groups to 128.45 µm (100 mV/cm), 149.39 µm (200 mV/cm), and 141.48 µm (400 mV/cm) in aligned fiber groups with electrical stimulation demonstrates the beneficial impact of electrical stimulation on nerve development (Fig. 6D). Electrical stimulation induces cell membrane depolarization on aligned fibers, enhancing the electrical charge in PPy nanoparticles. This leads to the accumulation of adhesive receptors, actin expansion in filopodia, and an increase in growth cones [24]. While actin concentration and self-assembly on one side of the cell dictate neurites/axons directional growth and cell orientation, the main impact of electrical stimulation is the promotion of cell adhesion, which negatively affects their migration. However, Zhang J et al*.* [53] showed that combining PCL/CNT aligned fibers with electrical stimulation (20 Hz and 100 mV) in the nerve conduit improves the directional growth of neurites/axons and cell orientation more effectively. This also enhances parameters like SFI, amplitude, latency, the number of axons, and myelin sheet thickness in sciatic nerve regeneration, similar to the gold standard autograft. Recently, instead of using electrical stimulation, which faces the challenge of reducing migration due to higher induction of adhesion foci, the use of piezoelectric polymers such as aligned PVDF fibers [125] is of interest. In this regard, after loading 400 nm PLLA nanofibers obtained by electrospinning (Flow rate: 0.5 mL/h, voltage: 12 kV) on PLLA films, Jiang F et al*.* [126] demonstrated that piezoelectric stimulation (~ 260 mV), not only enhanced cell differentiation (11% more than the non-stimulated group), but also led to a twofold increase in cell length compared to the non-stimulated group (142.7 µm vs. 70.2 µm). It was revealed that piezoelectric stimulation through double activation of Ca^2+^ channels initiates downstream signaling by increasing intracellular calcium. However, piezoelectric stimulation did not significantly affect cell orientation; instead, cell orientation was primarily determined by aligned fibers.

## Challenges and future perspective

Management of PNI treatment remains inadequate despite advances in drugs and surgical techniques. Reports indicate that fewer than 25% of patients undergoing nerve repair achieve optimal sensory and motor function recovery after 5 years. The scientific community is focused on improving nerve cell growth and guidance through new technologies to create conduits to overcome treatment obstacles. Utilizing conduits significantly enhances repair processes for damaged peripheral nerves by promoting nerve cell growth, neurite and axon directional growth, and nerve cell orientation. However, challenges exist in using conduits in medical settings, including:

- One of the main challenges in using conduits is ensuring biological safety in humans. A thorough comprehension of cell behaviors within conduits, as well as the behavior of nanoparticles loaded and released from conduits, can only be assessed through cell viability, migration, growth, proliferation, adhesion, and differentiation in short-term laboratory settings. The fluctuating nature of conduit degradation in the body, the range of inflammatory responses, the variety of cells affected, the extended human treatment process, the diverse immune system components, patient lifestyle, and medical history all contribute to the challenge of ensuring optimal conduit function. Thus, considering the constraints of the human model, focusing on models such as human organoids that closely resemble original tissue may aid in predicting potential immunogenic strategies.
- Conduit studies typically aim to promote the directional growth of neurons and align them during development or differentiation. However, the challenge of linearly distributing conductive materials and biological/chemical gradients hinders the targeted transmission of electrical-neural signals and the induction of directional growth of nerve cells, posing a significant issue for peripheral nerve repair. Focusing on accurately regulating the reception of electric-nerve pulses from the proximal end to the distal stump in conduits, based on determining the precise path of conductive material loading, can offer a clearer perspective on repairing damaged peripheral nerves. Additionally, relying solely on the detection of gradients in conduits is concerning. Thus, focusing on the extent of arrangmenet and diffusion of gradients, evaluating the specific quantity and quality of gradients, and considering their potential side effects on other parts or cells can offer valuable insights for their application.
- Despite the wide range of conduits available (biological, natural, synthetic), they are not yet a dependable choice for axon growth and nerve tissue regeneration over long distances, unlike autologous nerve grafts. To achieve this goal, a thorough comprehension of the physicochemical characteristics of conduits larger than 2.5 cm is essential, particularly in motor-sensory models resembling humans.
- In vitro studies and clinical trials have been widely conducted on rats, rabbits, dogs, monkeys, pigs, etc. While the generalization of their results to humans based on the complete non-compliance of the aspects of the biology of the repairing nerve, the path of research has become a concern. Focusing on human trials or human organoids that closely resemble original tissue can reduce some of the existing challenges.
- Conduits are typically designed and created by considering biocompatibility, biodegradability, enhancing cell adhesion, biomarker loading, creating topographical features, and adjusting mechanical properties for nerve tissue. However, the complexity and high cost of production methods pose challenges for commercialization. Focusing on cost-effective designs and simpler production techniques can facilitate their use in both general and personalized clinical applications.
- Conduit studies mainly rely on autologous cells to prevent transplant rejection, complicating the commercialization and treatment process. The pursuit of a universal neuron or neuron-like cell discovery will enhance the potential for clinical treatments.
- Conduits are commonly employed to direct axonal growth, inhibit fibrous tissue infiltration and scarring, and discourage axon reinnervation or nerve bundling. Prioritizing the development of the vascular network, promoting the movement of neural stem cells from neighboring areas, and averting uncontrolled swelling of hydrogels within the cavities can enhance treatment outcomes.

## Conclusion

Despite the inherent regeneration of peripheral nerves, many nerve injuries encounter challenges like prolonged recovery, inflammation, neuroma, and decreased tissue function. Tissue engineering aims to address these obstacles by incorporating nanotechnologies and cell science to mimic the supportive structures of the ECM. This enhances cell proliferation, migration, and orientation, facilitating the directional growth of neurites/axons. This review suggests that promoting cone growth in a specific direction, achieving even cell distribution through targeted migration, enhancing the longitudinal growth of neurites/axons within conduits, and controlling cell organization to mimic natural tissue structure could be promising strategies. Studies have shown that incorporating micro- or nano-patterns in conduits (such as aligned fibers, grooves, channels, pillars, pits, etc.) along with therapeutic approaches like electrical stimulation and nano-carriers can have a substantial impact on nerve cell behavior, ultimately aiding in the restoration of damaged peripheral nerve functions. Conduits with surface topographies play a significant role in guiding axonal growth and cell orientation, inhibiting fibrous tissue penetration, and preventing axon re-innervation during peripheral nerve regeneration. The utilization of conduits with aligned fibers > multi-channels > grooves has garnered considerable interest due to reduced manufacturing complexity, enhanced ability to direct growth and cell migration, improved accessibility, decreased nerve compression, and control over fiber diameter and myelin thickness. However, the mechanism of action of topography on the development and expansion of peripheral nerves is currently being elucidated. Therefore, thorough research is necessary to understand the mechanism of micro- or nano-patterns and cellular responses based on their physicochemical characteristics, enabling the development of clinical applications with greater confidence. In the following, the focus should be on understanding the mechanism of micro- or nano-patterns in nerve cells to predict cell orientation, neurite/axon growth, and tissue function restoration efficiently. Also, despite various conduit manufacturing techniques, establishing a specific standard or tactic for creating micro or nano patterns is essential to enhance their operational capability in nerve tissue reliably, while addressing issues such as construction complexity, high cost, low reproducibility, and solvent presence. Therefore, besides evaluating conduit surface size and resolution, it is vital to investigate the commercialization process of conduits through biomedical models. This implies that the manufacturing technique should account for the mechanical and chemical changes on conduit surfaces that affect cell migration and adhesion strength, mimicking cell behaviors in the ECM. Recent research indicates that cells develop more focal adhesions on patterned surfaces, which could potentially hinder cell migration and morphological expansion processes, going against their natural tendencies. Finally, this review demonstrates that creating micro- or nano-patterns in the nerve conduit can enhance the peripheral nerve regeneration process by improving motor performance in model mice and tissue structures. Surprisingly, this approach also matches the efficiency of nerve tissue regeneration seen with the gold standard of autograft.

## Data Availability

We have included 6 figures. For all of them, copyright permission from the copyright holder was necessary. We have mentioned this in the manuscript with proper citations.

## Abbreviations

3D: Three dimensional
CMAP: Compound muscle action potential
CNT: Carbon nanotube
DRG: Dorsal root ganglion
IL: Interleukin
IONPs: Iron oxide nanoparticles
MAPK/ERK: Mitogen-activated protein kinase/extracellular signal-regulated kinase
NCAD: Neural cadherin
NCAM: Neural cell adhesion molecule
NCV: Nerve conduction velocity
NGF: Nerve growth factor
PCL: Polycaprolactone
PDMS: Polydimethylsiloxane
PEG: Polyethylene glycol
PLCL: Poly(L-lactide-co-ε-caprolactone)
PLL: Poly-L-lysine
PLLA: Poly(L-lactide)
PLGA: Poly lactic-co-glycolic acid
PNI: Peripheral nerve injury
PPy: Polypyrrole
PTFE: Polytetrafluoroethylene
PVDF: Poly(vinylidene fluoride)
PU: Polyurethane
Sema3A: Semaphorin-3A
SF: Silk fibroin
SFI: Sciatic functional index
SPION: Superparamagnetic iron oxide nanoparticle
TNF: Tumor necrosis factor

## References

[1] Sharifi M, Farahani MK, Salehi M, Atashi A, Alizadeh M, Kheradmandi R, Molzemi S (2022). Exploring the physicochemical, electroactive, and biodelivery properties of metal nanoparticles on peripheral nerve regeneration. ACS Biomater Sci Eng.

[2] Qian Y, Lin H, Yan Z, Shi J, Fan C (2021). Functional nanomaterials in peripheral nerve regeneration: scaffold design, chemical principles and microenvironmental remodeling. Mater Today.

[3] Liu F, Xu J, Wu L, Zheng T, Han Q, Liang Y, Zhang L, Li G, Yang Y (2021). The influence of the surface topographical cues of biomaterials on nerve cells in peripheral nerve regeneration: a review. Stem Cells Int.

[4] Kim SM, Lee MS, Jeon J, Lee DH, Yang K, Cho SW, Han I, Yang HS (2018). Biodegradable nerve guidance conduit with microporous and micropatterned poly (lactic-co-glycolic acid)-accelerated sciatic nerve regeneration. Macromol Biosci.

[5] Hu Y, Chen Z, Wang H, Guo J, Cai J, Chen X, Wei H, Qi J, Wang Q, Liu H (2022). Conductive nerve guidance conduits based on morpho butterfly wings for peripheral nerve repair. ACS Nano.

[6] Zheng T, Wu L, Xu J, Sun S, Guan W, Han Q, Zhang L, Gu X, Yang Y, Li G (2022). YR/DFO@DCNT functionalized anisotropic micro/nano composite topography scaffolds for accelerating long-distance peripheral nerve regeneration. Compos B Eng.

[7] Huang L, Gao J, Wang H, Xia B, Yang Y, Xu F, Zheng X, Huang J, Luo Z (2020). Fabrication of 3D scaffolds displaying biochemical gradients along longitudinally oriented microchannels for neural tissue engineering. ACS Appl Mater Interfaces.

[8] Huang Z, Sun M, Li Y, Guo Z, Li H (2021). Reduced graphene oxide-coated electrospun fibre: effect of orientation, coverage and electrical stimulation on Schwann cells behavior. J Mater Chem B.

[9] Ma Y, Gao H, Wang H, Cao X (2021). Engineering topography: effects on nerve cell behaviors and applications in peripheral nerve repair. J Mater Chem B.

[10] Rigby MJ, Gomez TM, Puglielli L (2020). Glial cell-axonal growth cone interactions in neurodevelopment and regeneration. Front Neurosci.

[11] Kozulin P, Richards LJ, Pfaff DW, Volkow ND, Rubenstein JL (2022). Axonal guidance: making connections. Neuroscience in the 21st century: from basic to clinical.

[12] Dravid A, O’Carroll SJ, Svirskis D, Rajendram R, Preedy VR, Martin CR (2022). Neurotrophins and their role in axonal outgrowth following spinal cord injury. Cellular, molecular, physiological, and behavioral aspects of spinal cord injury.

[13] SenGupta S, Parent CA, Bear JE (2021). The principles of directed cell migration. Nat Rev Mol Cell Biol.

[14] Oh B, Wu YW, Swaminathan V, Lam V, Ding J, George PM (2021). Modulating the electrical and mechanical microenvironment to guide neuronal stem cell differentiation. Adv Sci.

[15] Chu X-L, Song X-Z, Li Q, Li Y-R, He F, Gu X-S, Ming D (2022). Basic mechanisms of peripheral nerve injury and treatment via electrical stimulation. Neural Regen Res.

[16] Song S, McConnell KW, Amores D, Levinson A, Vogel H, Quarta M, Rando TA, George PM (2021). Electrical stimulation of human neural stem cells via conductive polymer nerve guides enhances peripheral nerve recovery. Biomaterials.

[17] Bierman-Duquette RD, Safarians G, Huang J, Rajput B, Chen JY, Wang ZZ, Seidlits SK (2022). Engineering tissues of the central nervous system: interfacing conductive biomaterials with neural stem/progenitor cells. Adv Healthcare Mater.

[18] Thrivikraman G, Boda SK, Basu B (2018). Unraveling the mechanistic effects of electric field stimulation towards directing stem cell fate and function: a tissue engineering perspective. Biomaterials.

[19] Musselman ED, Cariello JE, Grill WM, Pelot NA (2021). ASCENT (automated simulations to characterize electrical nerve thresholds): a pipeline for sample-specific computational modeling of electrical stimulation of peripheral nerves. PLoS Comput Biol.

[20] Eftekhari BS, Eskandari M, Janmey PA, Samadikuchaksaraei A, Gholipourmalekabadi M (2020). Surface topography and electrical signaling: single and synergistic effects on neural differentiation of stem cells. Adv Func Mater.

[21] Repić T, Madirazza K, Bektur E, Sapunar D (2016). Characterization of dorsal root ganglion neurons cultured on silicon micro-pillar substrates. Sci Rep.

[22] Srinivasan A, Tahilramani M, Bentley JT, Gore RK, Millard DC, Mukhatyar VJ, Joseph A, Haque AS, Stanley GB, English AW, Bellamkonda RV (2015). Microchannel-based regenerative scaffold for chronic peripheral nerve interfacing in amputees. Biomaterials.

[23] Oh SH, Kang JG, Kim TH, Namgung U, Song KS, Jeon BH, Lee JH (2018). Enhanced peripheral nerve regeneration through asymmetrically porous nerve guide conduit with nerve growth factor gradient. J Biomed Mater Res, Part A.

[24] Zou Y, Qin J, Huang Z, Yin G, Pu X, He D (2016). Fabrication of aligned conducting PPy-PLLA fiber films and their electrically controlled guidance and orientation for neurites. ACS Appl Mater Interfaces.

[25] Lu K, Qian Y, Gong J, Zhu Z, Yin J, Ma L, Yu M, Wang H (2021). Biofabrication of aligned structures that guide cell orientation and applications in tissue engineering. Bio-Des Manuf.

[26] Antoniadis G, Haastert-Talini K, Assmus H, Antoniadis G (2017). The peripheral nerve: neuroanatomical principles before and after injury. Modern concepts of peripheral nerve repair.

[27] Reina MA, Boezaart AP, Tubbs RS, Zasimovich Y, Fernández-Domínguez M, Fernández P, Sala-Blanch X (2020). Another (internal) epineurium: beyond the anatomical barriers of nerves. Clin Anat.

[28] Pestronk A, Schmidt RE, Bucelli R, Sim J (2023). Schwann cells and myelin in human peripheral nerve: major protein components vary with age, axon size and pathology. Neuropathol Appl Neurobiol.

[29] Wilson ER, Della-Flora Nunes G, Weaver MR, Frick LR, Feltri ML (2021). Schwann cell interactions during the development of the peripheral nervous system. Dev Neurobiol.

[30] Papagiannis G, Triantafyllou A, Stasi S, Yiannopoulou KG, Papathanasiou G, Mitsiokapa E, Papadopoulos EC, Papagelopoulos PJ, Koulouvaris P (2020). Biomechanical behavior and viscoelastic properties of peripheral nerves subjected to tensile stress: common injuries and current repair techniques. Crit Rev Phys Rehab Med.

[31] Chooi WH, Chew SY (2019). Modulation of cell-cell interactions for neural tissue engineering: potential therapeutic applications of cell adhesion molecules in nerve regeneration. Biomaterials.

[32] Gärtner A, Fornasiero EF, Dotti CG (2015). Cadherins as regulators of neuronal polarity. Cell Adh Migr.

[33] Guan X, Guan X, Dong C, Jiao Z (2020). Rho GTPases and related signaling complexes in cell migration and invasion. Exp Cell Res.

[34] Sowparani S, Mahalakshmi P, Sweety JP, Francis AP, Dhanalekshmi U, Selvasudha N (2022). Ubiquitous neural cell adhesion molecule (NCAM): potential mechanism and valorisation in cancer pathophysiology, drug targeting and molecular transductions. Mol Neurobiol.

[35] Kataria H, Alizadeh A, Karimi-Abdolrezaee S (2019). Neuregulin-1/ErbB network: an emerging modulator of nervous system injury and repair. Prog Neurobiol.

[36] Han G-H, Peng J, Liu P, Ding X, Wei S, Lu S, Wang Y (2019). Therapeutic strategies for peripheral nerve injury: decellularized nerve conduits and Schwann cell transplantation. Neural Regen Res.

[37] Liu F, Xu J, Wu L, Zheng T, Han Q, Liang Y, Zhang L, Li G, Yang Y (2021). The influence of the surface topographical cues of biomaterials on nerve cells in peripheral nerve regeneration: a review. Stem Cells Int.

[38] Romano NH, Madl CM, Heilshorn SC (2015). Matrix RGD ligand density and L1CAM-mediated Schwann cell interactions synergistically enhance neurite outgrowth. Acta Biomater.

[39] Stukel JM, Willits RK (2016). Mechanotransduction of neural cells through cell–substrate interactions. Tissue Eng Part B Rev.

[40] Goldmann WH (2016). Role of vinculin in cellular mechanotransduction. Cell Biol Int.

[41] Wang H, Wang X, Qu J, Yue Q, Yn Hu, Zhang H (2015). VEGF enhances the migration of MSCs in neural differentiation by regulating focal adhesion turnover. J Cell Physiol.

[42] Zhang H, Guo J, Wang Y, Shang L, Chai R, Zhao Y (2022). Natural polymer-derived bioscaffolds for peripheral nerve regeneration. Adv Func Mater.

[43] Gregory H, Phillips JB (2021). Materials for peripheral nerve repair constructs: natural proteins or synthetic polymers?. Neurochem Int.

[44] Riccio M, Marchesini A, Pugliese P, De Francesco F (2019). Nerve repair and regeneration: biological tubulization limits and future perspectives. J Cell Physiol.

[45] Heinzel JC, Quyen Nguyen M, Kefalianakis L, Prahm C, Daigeler A, Hercher D, Kolbenschlag J (2021). A systematic review and meta-analysis of studies comparing muscle-in-vein conduits with autologous nerve grafts for nerve reconstruction. Sci Rep.

[46] Sun P, Guan Y, Yang C, Hou H, Liu S, Yang B, Li X, Chen S, Wang L, Wang H (2023). A bioresorbable and conductive scaffold integrating silicon membranes for peripheral nerve regeneration. Adv Healthcare Mater.

[47] Redondo-Gomez C, Leandro-Mora R, Blanch-Bermudez D, Espinoza-Araya C, Hidalgo-Barrantes D, Vega-Baudrit J (2020). Recent advances in carbon nanotubes for nervous tissue regeneration. Adv Polym Technol.

[48] Sharifi M, Kheradmandi R, Salehi M, Alizadeh M, ten Hagen TLM, Falahati M (2022). Criteria, challenges, and opportunities for acellularized allogeneic/xenogeneic bone grafts in bone repairing. ACS Biomater Sci Eng.

[49] Jeong H-J, Nam H, Jang J, Lee S-J (2020). 3D bioprinting strategies for the regeneration of functional tubular tissues and organs. Bioengineering.

[50] Askari M, Naniz MA, Kouhi M, Saberi A, Zolfagharian A, Bodaghi M (2021). Recent progress in extrusion 3D bioprinting of hydrogel biomaterials for tissue regeneration: a comprehensive review with focus on advanced fabrication techniques. Biomater Sci.

[51] Yan Y, Yao R, Zhao J, Chen K, Duan L, Wang T, Zhang S, Guan J, Zheng Z, Wang X (2022). Implantable nerve guidance conduits: material combinations, multi-functional strategies and advanced engineering innovations. Bioactive Mater.

[52] Sarker M, Naghieh S, McInnes AD, Schreyer DJ, Chen X (2018). Strategic design and fabrication of nerve guidance conduits for peripheral nerve regeneration. Biotechnol J.

[53] Zhang J, Zhang X, Wang C, Li F, Qiao Z, Zeng L, Wang Z, Liu H, Ding J, Yang H (2021). Conductive composite fiber with optimized alignment guides neural regeneration under electrical stimulation. Adv Healthcare Mater.

[54] Du J, Liu J, Yao S, Mao H, Peng J, Sun X, Cao Z, Yang Y, Xiao B, Wang Y (2017). Prompt peripheral nerve regeneration induced by a hierarchically aligned fibrin nanofiber hydrogel. Acta Biomater.

[55] Singh A, Asikainen S, Teotia AK, Shiekh PA, Huotilainen E, Qayoom I, Partanen J, Seppälä J, Kumar A (2018). Biomimetic photocurable three-dimensional printed nerve guidance channels with aligned cryomatrix lumen for peripheral nerve regeneration. ACS Appl Mater Interfaces.

[56] Zheng C, Yang Z, Chen S, Zhang F, Rao Z, Zhao C, Quan D, Bai Y, Shen J (2021). Nanofibrous nerve guidance conduits decorated with decellularized matrix hydrogel facilitate peripheral nerve injury repair. Theranostics.

[57] Lu Q, Zhang F, Cheng W, Gao X, Ding Z, Zhang X, Lu Q, Kaplan DL (2021). Nerve guidance conduits with hierarchical anisotropic architecture for peripheral nerve regeneration. Adv Healthcare Mater.

[58] Kim JI, Hwang TI, Aguilar LE, Park CH, Kim CS (2016). A controlled design of aligned and random nanofibers for 3D bi-functionalized nerve conduits fabricated via a novel electrospinning set-up. Sci Rep.

[59] Chen S, Du Z, Zou J, Qiu S, Rao Z, Liu S, Sun X, Xu Y, Zhu Q, Liu X (2019). Promoting neurite growth and schwann cell migration by the harnessing decellularized nerve matrix onto nanofibrous guidance. ACS Appl Mater Interfaces.

[60] Quan Q, Meng H-Y, Chang B, Liu G-B, Cheng X-Q, Tang H, Wang Y, Peng J, Zhao Q, Lu S-B (2019). Aligned fibers enhance nerve guide conduits when bridging peripheral nerve defects focused on early repair stage. Neural Regen Res.

[61] Hu N, Wu H, Xue C, Gong Y, Wu J, Xiao Z, Yang Y, Ding F, Gu X (2013). Long-term outcome of the repair of 50 mm long median nerve defects in rhesus monkeys with marrow mesenchymal stem cells-containing, chitosan-based tissue engineered nerve grafts. Biomaterials.

[62] Dong X, Yang Y, Bao Z, Midgley AC, Li F, Dai S, Yang Z, Wang J, Liu L, Li W (2023). Micro-nanofiber composite biomimetic conduits promote long-gap peripheral nerve regeneration in canine models. Bioactive Mater.

[63] Wang L, Wu Y, Hu T, Ma PX, Guo B (2019). Aligned conductive core-shell biomimetic scaffolds based on nanofiber yarns/hydrogel for enhanced 3D neurite outgrowth alignment and elongation. Acta Biomater.

[64] Zhang Z, Jørgensen ML, Wang Z, Amagat J, Wang Y, Li Q, Dong M, Chen M (2020). 3D anisotropic photocatalytic architectures as bioactive nerve guidance conduits for peripheral neural regeneration. Biomaterials.

[65] Dong X, Liu S, Yang Y, Gao S, Li W, Cao J, Wan Y, Huang Z, Fan G, Chen Q (2021). Aligned microfiber-induced macrophage polarization to guide schwann-cell-enabled peripheral nerve regeneration. Biomaterials.

[66] Muangsanit P, Roberton V, Costa E, Phillips JB (2021). Engineered aligned endothelial cell structures in tethered collagen hydrogels promote peripheral nerve regeneration. Acta Biomater.

[67] Fan J, Zhang Y, Liu Y, Wang Y, Cao F, Yang Q, Tian F (2019). Explanation of the cell orientation in a nanofiber membrane by the geometric potential theory. Results Phys.

[68] Simitzi C, Ranella A, Stratakis E (2017). Controlling the morphology and outgrowth of nerve and neuroglial cells: the effect of surface topography. Acta Biomater.

[69] Yang K, Jung K, Ko E, Kim J, Park KI, Kim J, Cho S-W (2013). Nanotopographical manipulation of focal adhesion formation for enhanced differentiation of human neural stem cells. ACS Appl Mater Interfaces.

[70] Baek J, Cho S-Y, Kang H, Ahn H, Jung W-B, Cho Y, Lee E, Cho S-W, Jung H-T, Im SG (2018). Distinct mechanosensing of human neural stem cells on extremely limited anisotropic cellular contact. ACS Appl Mater Interfaces.

[71] Cai L, Zhang L, Dong J, Wang S (2012). Photocured biodegradable polymer substrates of varying stiffness and microgroove dimensions for promoting nerve cell guidance and differentiation. Langmuir.

[72] Liguori GR, Zhou Q, Liguori TTA, Barros GG, Kühn PT, Moreira LFP, Van Rijn P, Harmsen MC (2019). Directional topography influences adipose mesenchymal stromal cell plasticity: prospects for tissue engineering and fibrosis. Stem Cells Int.

[73] Tonazzini I, Jacchetti E, Meucci S, Beltram F, Cecchini M (2015). Schwann cell contact guidance versus boundary ­interaction in functional wound healing along nano and microstructured membranes. Adv Healthcare Mater.

[74] Huang C, Ouyang Y, Niu H, He N, Ke Q, Jin X, Li D, Fang J, Liu W, Fan C, Lin T (2015). Nerve guidance conduits from aligned nanofibers: improvement of nerve regeneration through longitudinal nanogrooves on a fiber surface. ACS Appl Mater Interfaces.

[75] Polo Y, Luzuriaga J, Iturri J, Irastorza I, Toca-Herrera JL, Ibarretxe G, Unda F, Sarasua J-R, Pineda JR, Larrañaga A (2021). Nanostructured scaffolds based on bioresorbable polymers and graphene oxide induce the aligned migration and accelerate the neuronal differentiation of neural stem cells. Nanomed: Nanotechnol, Biol Med.

[76] Zhang D, Yao Y, Duan Y, Yu X, Shi H, Nakkala JR, Zuo X, Hong L, Mao Z, Gao C (2020). Surface-anchored graphene oxide nanosheets on cell-scale micropatterned poly(d, l-lactide-co-caprolactone) conduits promote peripheral nerve regeneration. ACS Appl Mater Interfaces.

[77] Li G, Zheng T, Wu L, Han Q, Lei Y, Xue L, Zhang L, Gu X, Yang Y (2021). Bionic microenvironment-inspired synergistic effect of anisotropic micro-nanocomposite topology and biology cues on peripheral nerve regeneration. Sci Adv.

[78] Lu S, Chen W, Wang J, Guo Z, Xiao L, Wei L, Yu J, Yuan Y, Chen W, Bian M (2023). Polydopamine-decorated plcl conduit to induce synergetic effect of electrical stimulation and topological morphology for peripheral nerve regeneration. Small Methods.

[79] Zheng T, Wu L, Sun S, Xu J, Han Q, Liu Y, Wu R, Li G (2022). Co-culture of Schwann cells and endothelial cells for synergistically regulating dorsal root ganglion behavior on chitosan-based anisotropic topology for peripheral nerve regeneration. Burns Trauma.

[80] Gu X, Chen X, Tang X, Zhou Z, Huang T, Yang Y, Ling J (2021). Pure-silk fibroin hydrogel with stable aligned micropattern toward peripheral nerve regeneration. Nanotechnol Rev.

[81] Mobasseri A, Faroni A, Minogue BM, Downes S, Terenghi G, Reid AJ (2015). Polymer scaffolds with preferential parallel grooves enhance nerve regeneration. Tissue Eng Part A.

[82] Scaccini L, Mezzena R, De Masi A, Gagliardi M, Gambarotta G, Cecchini M, Tonazzini I (2021). Chitosan micro-grooved membranes with increased asymmetry for the improvement of the schwann cell response in nerve regeneration. Int J Mol Sci.

[83] Zhang D, Xu S, Wu S, Gao C (2018). Micropatterned poly(d, l-lactide-co-caprolactone) films entrapped with gelatin for promoting the alignment and directional migration of Schwann cells. J Mater Chem B.

[84] Zhang D, Wu S, Feng J, Duan Y, Xing D, Gao C (2018). Micropatterned biodegradable polyesters clicked with CQAASIKVAV promote cell alignment, directional migration, and neurite outgrowth. Acta Biomater.

[85] Wang J, Xiong H, Zhu T, Liu Y, Pan H, Fan C, Zhao X, Lu WW (2020). Bioinspired multichannel nerve guidance conduit based on shape memory nanofibers for potential application in peripheral nerve repair. ACS Nano.

[86] You R, Zhang Q, Li X, Yan S, Luo Z, Qu J, Li M (2020). Multichannel bioactive silk nanofiber conduits direct and enhance axonal regeneration after spinal cord injury. ACS Biomater Sci Eng.

[87] Jiang Z, Zhang Y, Wang Y, Wang S, Chang J, Liu W, Han B (2023). Multichannel nerve conduit based on chitosan derivates for peripheral nerve regeneration and schwann cell survival. Carbohyd Polym.

[88] Ye W, Li H, Yu K, Xie C, Wang P, Zheng Y, Zhang P, Xiu J, Yang Y, Zhang F (2020). 3D printing of gelatin methacrylate-based nerve guidance conduits with multiple channels. Mater Des.

[89] Ankam S, Suryana M, Chan LY, Moe AAK, Teo BK, Law JB, Sheetz MP, Low HY, Yim EK (2013). Substrate topography and size determine the fate of human embryonic stem cells to neuronal or glial lineage. Acta Biomater.

[90] Lunghi A, Mariano A, Bianchi M, Dinger NB, Murgia M, Rondanina E, Toma A, Greco P, Di Lauro M, Santoro F (2022). Flexible neural interfaces based on 3D PEDOT:PSS micropillar arrays. Adv Mater Interfaces.

[91] Vinzons LU, Lin S-P (2022). Hierarchical micro-/nanotopographies patterned by tandem nanosphere lens lithography and uv–led photolithography for modulating pc12 neuronal differentiation. ACS Applied Nano Mater.

[92] Bucaro MA, Vasquez Y, Hatton BD, Aizenberg J (2012). Fine-tuning the degree of stem cell polarization and alignment on ordered arrays of high-aspect-ratio nanopillars. ACS Nano.

[93] Tse K-H, Sun M, Mantovani C, Terenghi G, Downes S, Kingham PJ (2010). In vitro evaluation of polyester-based scaffolds seeded with adipose derived stem cells for peripheral nerve regeneration. J Biomed Mater Res, Part A.

[94] Zhu M, Li W, Dong X, Yuan X, Midgley AC, Chang H, Wang Y, Wang H, Wang K, Ma PX (2019). In vivo engineered extracellular matrix scaffolds with instructive niches for oriented tissue regeneration. Nat Commun.

[95] Rao Z, Lin T, Qiu S, Zhou J, Liu S, Chen S, Wang T, Liu X, Zhu Q, Bai Y, Quan D (2021). Decellularized nerve matrix hydrogel scaffolds with longitudinally oriented and size-tunable microchannels for peripheral nerve regeneration. Mater Sci Eng, C.

[96] Pawelec KM, Yoon C, Giger RJ, Sakamoto J (2019). Engineering a platform for nerve regeneration with direct application to nerve repair technology. Biomaterials.

[97] Park D, Kim D, Park SJ, Choi JH, Seo Y, Kim D-H, Lee S-H, Hyun JK, Yoo J, Jung Y (2022). Micropattern-based nerve guidance conduit with hundreds of microchannels and stem cell recruitment for nerve regeneration. NPJ Regen Med.

[98] Zhang K, Xiao X, Wang X, Fan Y, Li X (2019). Topographical patterning: characteristics of current processing techniques, controllable effects on material properties and co-cultured cell fate, updated applications in tissue engineering, and improvement strategies. J Mater Chem B.

[99] Kundu A, Micholt L, Friedrich S, Rand DR, Bartic C, Braeken D, Levchenko A (2013). Superimposed topographic and chemical cues synergistically guide neurite outgrowth. Lab Chip.

[100] Micholt L, Gärtner A, Prodanov D, Braeken D, Dotti CG, Bartic C (2013). Substrate topography determines neuronal polarization and growth in vitro. PLoS ONE.

[101] Fan S, Qi L, Li J, Pan D, Zhang Y, Li R, Zhang C, Wu D, Lau P, Hu Y (2021). Guiding the patterned growth of neuronal axons and dendrites using anisotropic micropillar scaffolds. Adv Healthcare Mater.

[102] Vedaraman S, Perez-Tirado A, Haraszti T, Gerardo-Nava J, Nishiguchi A, De Laporte L (2021). Anisometric microstructures to determine minimal critical physical cues required for neurite alignment. Adv Healthcare Mater.

[103] Baranes K, Chejanovsky N, Alon N, Sharoni A, Shefi O (2012). Topographic cues of nano-scale height direct neuronal growth pattern. Biotechnol Bioeng.

[104] Milos F, Belu A, Mayer D, Maybeck V, Offenhäusser A (2021). Polymer nanopillars induce increased paxillin adhesion assembly and promote axon growth in primary cortical neurons. Adv Biol.

[105] Milos F, Tullii G, Gobbo F, Lodola F, Galeotti F, Verpelli C, Mayer D, Maybeck V, Offenhäusser A, Antognazza MR (2021). High aspect ratio and light-sensitive micropillars based on a semiconducting polymer optically regulate neuronal growth. ACS Appl Mater Interfaces.

[106] Xu X, Wang W, Kratz K, Fang L, Li Z, Kurtz A, Ma N, Lendlein A (2014). Controlling major cellular processes of human mesenchymal stem cells using microwell structures. Adv Healthcare Mater.

[107] Mobasseri S, Terenghi G, Downes S (2013). Micro-structural geometry of thin films intended for the inner lumen of nerve conduits affects nerve repair. J Mater Sci: Mater Med.

[108] Yang A, Huang Z, Yin G, Pu X (2015). Fabrication of aligned, porous and conductive fibers and their effects on cell adhesion and guidance. Colloids Surf, B.

[109] Zhang Z, Wang Y, Chen Z, Xu D, Zhang D, Wang F, Zhao Y (2022). Tailoring conductive inverse opal films with anisotropic elliptical porous patterns for nerve cell orientation. J Nanobiotechnol.

[110] Piscioneri A, Morelli S, Ritacco T, Giocondo M, Peñaloza R, Drioli E, De Bartolo L (2023). Topographical cues of PLGA membranes modulate the behavior of hMSCs, myoblasts and neuronal cells. Colloids Surf, B.

[111] Kim JA, Lee N, Kim BH, Rhee WJ, Yoon S, Hyeon T, Park TH (2011). Enhancement of neurite outgrowth in PC12 cells by iron oxide nanoparticles. Biomaterials.

[112] Seo J, Kim J, Joo S, Choi JY, Kang K, Cho WK, Choi IS (2018). Nanotopography-promoted formation of axon collateral branches of hippocampal neurons. Small.

[113] Cho Y, Choi Y, Seong H (2024). Nanoscale surface coatings and topographies for neural interfaces. Acta Biomater.

[114] Antman-Passig M, Shefi O (2016). Remote magnetic orientation of 3d collagen hydrogels for directed neuronal regeneration. Nano Lett.

[115] Antman-Passig M, Giron J, Karni M, Motiei M, Schori H, Shefi O (2021). Magnetic assembly of a multifunctional guidance conduit for peripheral nerve repair. Adv Func Mater.

[116] Xia L, Zhang C, Su K, Fan J, Niu Y, Yu Y, Chai R (2022). Oriented growth of neural stem cell-derived neurons regulated by magnetic nanochains. Front Bioeng Biotechnol.

[117] Rose JC, Cámara-Torres M, Rahimi K, Köhler J, Möller M, De Laporte L (2017). Nerve cells decide to orient inside an injectable hydrogel with minimal structural guidance. Nano Lett.

[118] Omidinia-Anarkoli A, Boesveld S, Tuvshindorj U, Rose JC, Haraszti T, De Laporte L (2017). An injectable hybrid hydrogel with oriented short fibers induces unidirectional growth of functional nerve cells. Small.

[119] Alsmadi NZ, Patil LS, Hor EM, Lofti P, Razal JM, Chuong C-J, Wallace GG, Romero-Ortega MI (2015). Coiled polymeric growth factor gradients for multi-luminal neural chemotaxis. Brain Res.

[120] Hsu RS, Chen PY, Fang JH, Chen YY, Chang CW, Lu YJ, Hu SH (2019). Adaptable microporous hydrogels of propagating NGF-gradient by injectable building blocks for accelerated axonal outgrowth. Advanced Science.

[121] Zhu L, Jia S, Liu T, Yan L, Huang D, Wang Z, Chen S, Zhang Z, Zeng W, Zhang Y (2020). Aligned PCL fiber conduits immobilized with nerve growth factor gradients enhance and direct sciatic nerve regeneration. Adv Func Mater.

[122] Zhang D, Li Z, Shi H, Yao Y, Du W, Lu P, Liang K, Hong L, Gao C (2022). Micropatterns and peptide gradient on the inner surface of a guidance conduit synergistically promotes nerve regeneration in vivo. Bioactive Mater.

[123] Bertucci C, Koppes R, Dumont C, Koppes A (2019). Neural responses to electrical stimulation in 2D and 3D in vitro environments. Brain Res Bull.

[124] Zhu R, Sun Z, Li C, Ramakrishna S, Chiu K, He L (2019). Electrical stimulation affects neural stem cell fate and function in vitro. Exp Neurol.

[125] Liu Z, Cai M, Zhang X, Yu X, Wang S, Wan X, Wang ZL, Li L (2021). Cell-traction-triggered on-demand electrical stimulation for neuron-like differentiation. Adv Mater.

[126] Jiang F, Shan Y, Tian J, Xu L, Li C, Yu F, Cui X, Wang C, Li Z, Ren K (2023). Poly(l-Lactic Acid) nanofiber-based multilayer film for the electrical stimulation of nerve cells. Adv Mater Interfaces.

